# Fibroblast-to-cardiomyocyte lactate shuttle modulates hypertensive cardiac remodelling

**DOI:** 10.1186/s13578-023-01098-0

**Published:** 2023-08-15

**Authors:** Tong Wei, Yuetong Guo, Chenglin Huang, Mengwei Sun, Bin Zhou, Jing Gao, Weili Shen

**Affiliations:** 1 Department of Cardiovascular Medicine, State Key Laboratory of Medical Genomics, Shanghai Key Laboratory of Hypertension, Shanghai Institute of Hypertension, Ruijin Hospital, Shanghai Jiao Tong University School of Medicine, Shanghai, 200025 China; 2 Department of Cardiology, Shanghai General Hospital, Shanghai Jiao Tong University School of Medicine, Shanghai, 200080 China; 3 Key Laboratory of State General Administration of Sport, Shanghai Research Institute of Sports Science, Shanghai, 200030 China; 4 New Cornerstone Science Laboratory, State Key Laboratory of Cell Biology, CAS Center for Excellence in Molecular Cell Science, Shanghai Institute of Biochemistry and Cell Biology, Chinese Academy of Sciences, University of Chinese Academy of Sciences, Shanghai, 200031 China

**Keywords:** GCN5L1, MPC2, MCT1, Metabolism shift, Lactate shuttle

## Abstract

**Background:**

Cardiac fibroblasts (CFs) and cardiomyocytes are the major cell populations in the heart. CFs not only support cardiomyocytes by producing extracellular matrix (ECM) but also assimilate myocardial nutrient metabolism. Recent studies suggest that the classical intercellular lactate shuttle may function in the heart, with lactate transported from CFs to cardiomyocytes. However, the underlying mechanisms regarding the generation and delivery of lactate from CFs to cardiomyocytes have yet to be explored.

**Results:**

In this study, we found that angiotensin II (Ang II) induced CFs differentiation into myofibroblasts that, driven by cell metabolism, then underwent a shift from oxidative phosphorylation to aerobic glycolysis. During this metabolic conversion, the expression of amino acid synthesis 5-like 1 (GCN5L1) was upregulated and bound to and acetylated mitochondrial pyruvate carrier 2 (MPC2) at lysine residue 19. Hyperacetylation of MPC2^k19^ disrupted mitochondrial pyruvate uptake and mitochondrial respiration. GCN5L1 ablation downregulated MPC2^K19^ acetylation, stimulated mitochondrial pyruvate metabolism, and inhibited glycolysis and lactate accumulation. In addition, myofibroblast-specific GCN5L1-knockout mice (GCN5L1^fl/fl^: Periostin-Cre) showed reduced myocardial hypertrophy and collagen content in the myocardium. Moreover, cardiomyocyte-specific monocarboxylate transporter 1 (MCT1)-knockout mice (MCT1^fl/fl^: Myh6-Cre) exhibited blocked shuttling of lactate from CFs to cardiomyocytes and attenuated Ang II-induced cardiac hypertrophy.

**Conclusions:**

Our findings suggest that GCN5L1-MPC2 signalling pathway alters metabolic patterns, and blocking MCT1 interrupts the fibroblast-to-cardiomyocyte lactate shuttle, which may attenuate cardiac remodelling in hypertension.

**Graphical Abstract:**

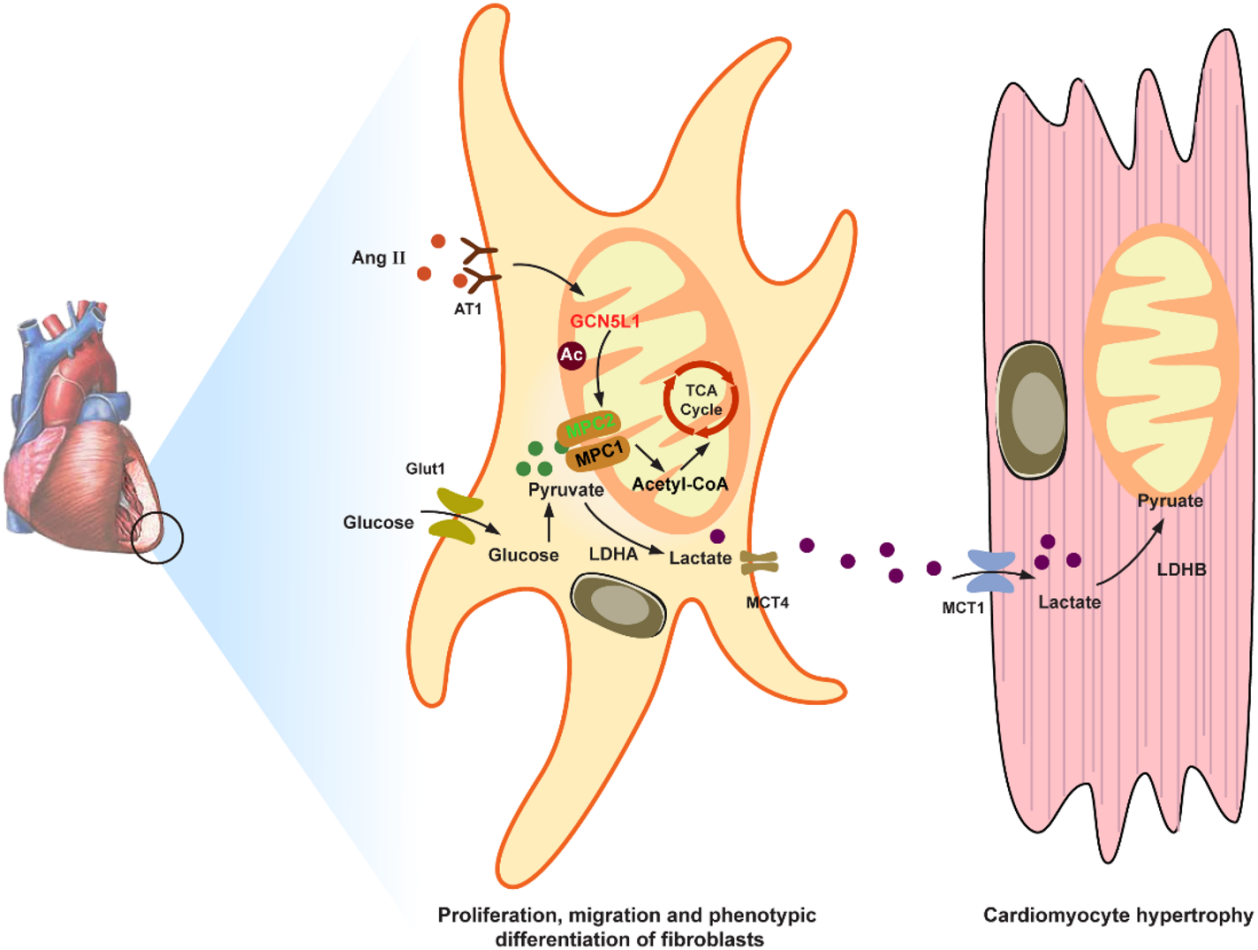

**Supplementary Information:**

The online version contains supplementary material available at 10.1186/s13578-023-01098-0.

## Highlights

1. Ang II elevated lactate production during CFs differentiation into myofibroblasts.

2. GCN5L1-mediated MPC2^K19^ acetylation reduces MPC activity.

3. Acetylation of MPC2^K19^ alters CFs metabolic patterns.

4. Blocking MCT1 activity disrupts the lactate shuttle and attenuates cardiomyocyte hypertrophy.

**Supplementary Information:**

The online version contains supplementary material available at 10.1186/s13578-023-01098-0.

## Introduction

Myocardial fibrosis and left ventricular hypertrophy are hallmarks of myocardial remodelling in hypertension [1]. Interactions between cardiomyocytes and fibroblasts play key roles in the regulation of cardiac remodelling under hypertensive stress; for example, they mediate paracrine signalling [2], mechanical stimulation [3], and extracellular matrix-mediated signalling [4]. Recent studies suggest that the classical intercellular lactate shuttle may function in the heart, where lactate is transported from fibroblasts to cardiomyocytes [5]. However, the underlying mechanisms regarding the generation and delivery of lactate from fibroblasts to cardiomyocytes remain unclear.

Differentiation of cardiac fibroblasts (CFs) into myofibroblasts greatly facilitates the initiation of myocardial fibrosis [6], a conversion process associated with an altered metabolic state [7]. Activated fibroblasts preferentially undergo glycolysis over mitochondrial oxidative phosphorylation, even under aerobic conditions [7]. This metabolic programming is regulated by mitochondrial pyruvate carrier (MPC), a hetero-oligomeric complex composed of MPC1 and MPC2 and is located on the inner mitochondrial membrane, where it transports pyruvate to the mitochondrial matrix for oxidative metabolism [8]. Impaired MPC activity enhances aerobic glycolysis, leading to cancer-associated fibroblast proliferation, migration, and collagen synthesis by providing lactate as a substrate for ATP production [9]. MPC overexpression is sufficient to inhibit cell proliferation because metabolism shifts from glycolysis to pyruvate oxidation [10]. In addition, MPC is involved in transcriptional and posttranslational modification networks. Previous studies have shown that hyperacetylation of MPC1 or MPC2 leads to MPC inactivation [11, 12]. Lysine acetylation is a posttranslational modification regulated by a lysine acetylase and deacetylase. The mitochondria-rich GCN5-like 1 (GCN5L1) protein is an important component of the mitochondrial acetyltransferase complex, which interacts with the deacetylase sirtuin-3 (SIRT3) to determine mitochondrial substrate activity [13]. It has been shown that SIRT3 knockdown leads to mitochondrial hyperacetylation, which is counteracted by simultaneous depletion of GCN5L1 [14]. Increasing evidence suggests that GCN5L1 is involved in regulating multiple mitochondrial biological functions, such as mitophagy, mitochondrial biogenesis, and fatty acid oxidation (FAO) [15–17]. There is compelling research indicating that GCN5L1 plays a critical role in regulating protein acetylation in the heart during maturation [18]. Further studies have confirmed that GCN5L1 plays a role in regulating energy substrate utilization and maintaining mitochondrial homeostasis in the adult heart [14, 19–21]. However, whether GCN5L1 mediates MPC acetylation and leads to CF exploitation of glycolysis is unclear.

The myocardium has a high energy requirement, and the cardiac metabolic network is flexible, depending on energy substrate availability. CFs have long been considered a negligible component in global cardiac energy metabolism. Intriguingly, previous studies showed that coculturing cardiomyocytes with CFs altered lactate transport protein activity in both cell types [22]. Several studies confirmed that fibroblast-derived lactate directly affects cardiomyocytes. Agnieszka et al. [22] suggested that exogenous lactate was associated with pathological hypertrophy and myocardial mechanical dysfunction. Conflicting data have shown that lactate causes cardiomyocyte dedifferentiation and proliferation, which may promote cardiac regeneration [23]. Although the effect of lactate on cardiomyocyte phenotype is debated, we hypothesize that to establish metabolic symbiosis with cardiomyocytes, CFs exploit glycolysis and engage in crosstalk with metabolic components in cardiomyocytes.

Angiotensin II (Ang II) is the major effector peptide of the renin-angiotensin system (RAS) and plays a key role in initiating CFs differentiation. In the present study, we investigated the role played by GCN5L1-mediated MPC acetylation in regulating metabolic reprogramming in CFs, which alters the metabolic pattern during CFs differentiation into myofibroblasts. Finally, we evaluated the effect of lactate accumulated in CFs that leads to an acidic microenvironment in the heart and alters the phenotype of neighbouring cardiomyocytes.

## Results

### Loss of GCN5L1 attenuates cardiac fibrosis and hypertrophy

**Fig. 1:**
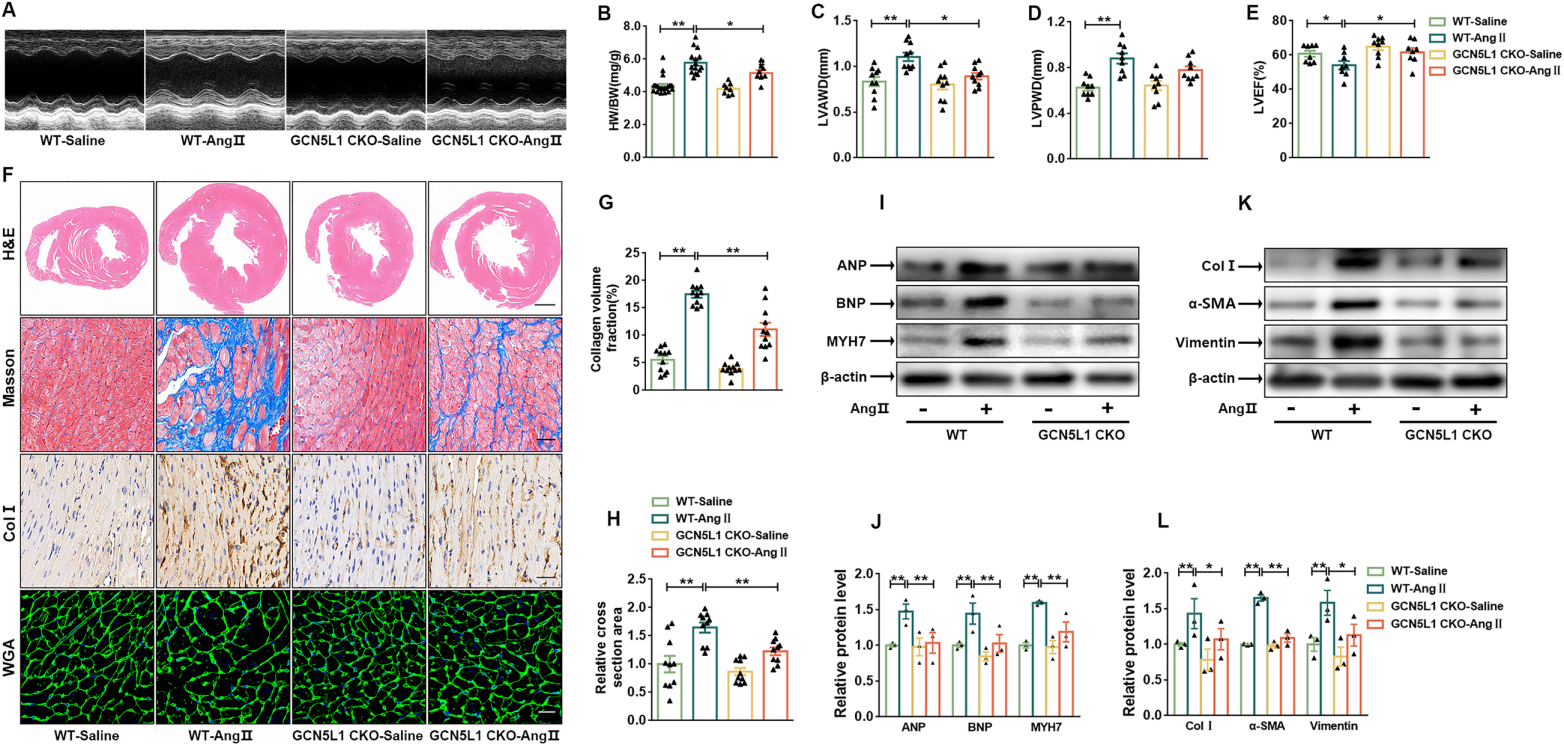
Loss of GCN5L1 attenuates cardiac fibrosis and hypertrophy. **A**, Echocardiographic analysis of WT and GCN5L1 CKO mice 4 weeks after saline or Ang II infusion. **B**, Heart weight/body weight (mg/g) ratio of four groups of mice(n ≥ 8/group). **C, D **and **E**, Quantification of echocardiography parameters LVAWD, LVPWD and LVEF(n ≥ 8/group). **F**, Representative images showing haematoxylin and eosin (H&E) staining (scale bar = 2 mm), Masson’s staining, immunohistology staining of Col I-positive cells in hearts and images showing WGA staining (scale bar = 100 μm). **G**, Quantification of collagen volume fraction(n = 11/group). **H**, Quantification of the relative cross-sectional area (n = 10/group). **I** and **J**, Representative immunoblot images showing ANP, BNP and MYH7 protein levels in hearts (**I**). The quantification of these proteins (**J**) (n = 3/group). **K** and **L**, Representative immunoblot images showing Col I, a-SMA and vimentin protein levels in hearts (**K**). The quantification of these proteins (**L**) (n = 3/group). The data are shown as the mean ± SEM. *P* values were calculated by one-way ANOVA. ^*^p < 0.05, ^**^p < 0.01

Postn is recognized as a marker of myofibroblasts expressed in regions of tissue damage [24]. To test the contribution of GCN5L1 to the regulation of cardiac fibrosis and hypertrophy, we generated mice lacking GCN5L1 expression in Postn-expressing myofibroblasts (GCN5L1 CKO) by crossing mice carrying floxed alleles of GCN5L1 (GCN5L1^fl/fl^) with Postn-CreERT2 mic. After 28 days of Ang II administration, western blot confirmed that the loss of GCN5L1 in cardiac myofibroblasts isolated from GCN5L1 CKO mice with Ang II infusion (Additional file 1: Fig. S1A). Although blood pressure was similarly increased in WT and GCN5L1 CKO mice during Ang II infusion (Additional file 1: Fig. S1B), the heart/body weight ratio (HW/BW) was found to be elevated in the WT mice, and this outcome was accompanied by an increase in LVAWD and LVPWD and a decreasing trend in LVEF. Myofibroblast-specific GCN5L1 deletion reversed Ang II-induced cardiac hypertrophy (Fig. 1A–E). In addition, a histological analysis revealed that Ang II-induced myocardial fibrosis and cardiomyocyte hypertrophy were significantly alleviated in the GCN5L1 CKO mice (Fig. 1F–H). This result was confirmed by western blot analysis, which showed a reduction in the product abundance of hypertrophy-related genes such as MYH7 (encoding MHC), ANP, and BNP (Fig. 1I–J) and of fibrosis-related genes such as collagen I, α-SMA and vimentin expression in the GCN5L1 CKO mice (Fig. 1K–L), suggesting that specific GCN5L1 deficiency in cardiac myofibroblasts attenuates Ang II-induced cardiac remodelling.

### GCN5L1 is critical for fibroblast-to-myofibroblast differentiation

**Fig. 2:**
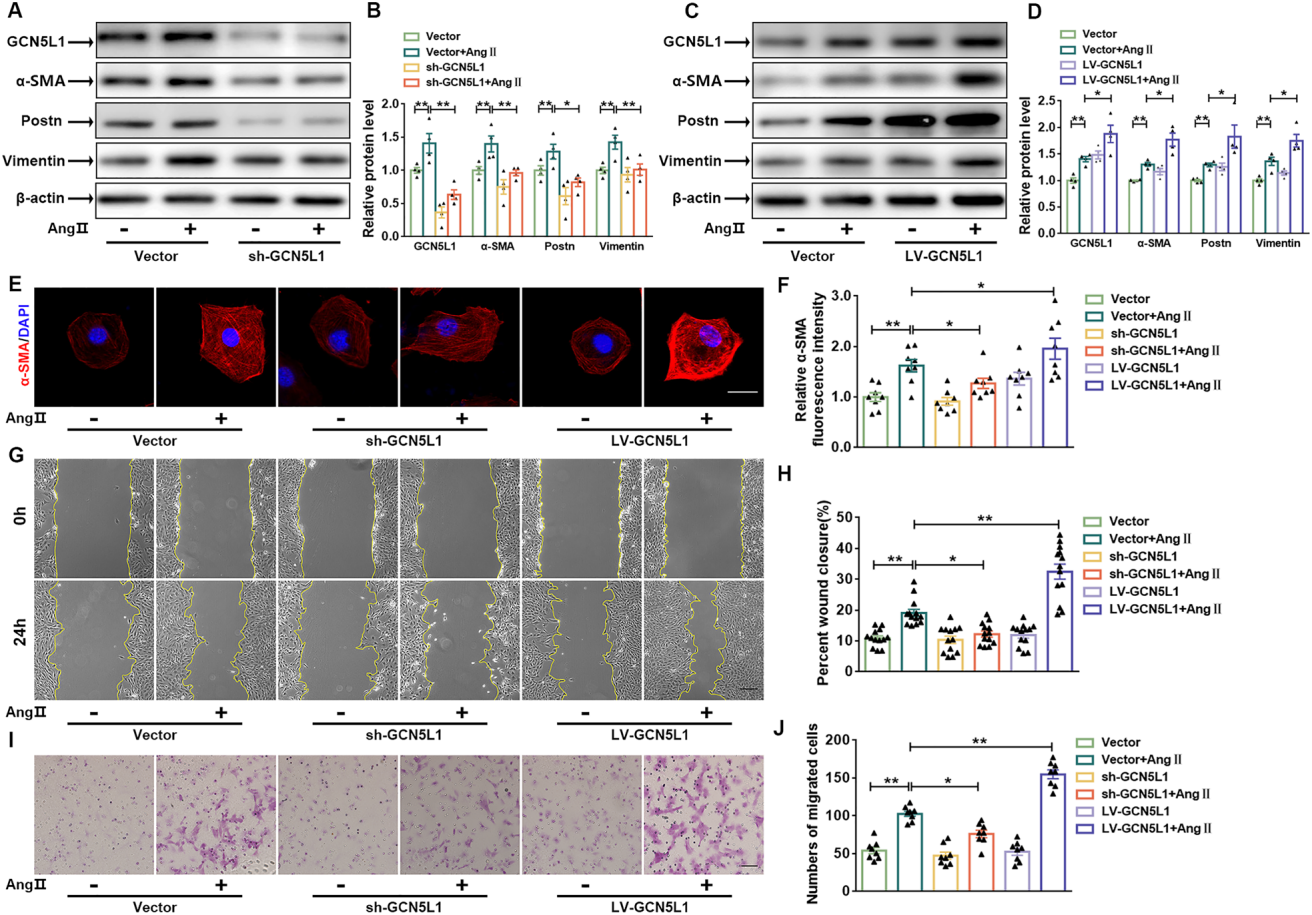
GCN5L1 is critical for the fibroblast-to-myofibroblast transition. **A** and **B** Representative images showing GCN5L1, a-SMA, Postn, and vimentin protein levels in CFs with or without Ang II treatment after transfection with an empty vector or GCN5L1-interfering lentiviruses (sh-GCN5L1) (**A**). The quantification of these proteins (**B**) (n = 4/group). **C** and **D** Representative immunoblot images showing GCN5L1, a-SMA, Postn and vimentin protein levels in CFs with or without Ang II after transfection with an empty vector or overexpressing lentiviruses (LV-GCN5L1) (**C**). The quantification of these proteins (**D**) (n = 4/group). **E** and **F** Representative images of a-SMA (red) and DAPI (4’,6-diamidino-2-phenylindole; blue) immunofluorescence. Scale bar = 100 μm (**E**). Quantification of the relative fluorescence intensity (**F**). **G** and **H** Representative images showing the cell wound healing assay (scale bar = 200 μm) (**G**) and quantification of the percentage of the wound closed (**H**) (n ≥ 8/group). **I** and **J** Representative images showing transwell migration and invasion experiments (scale bar = 200 μm) (**I**) and quantification of the number of migrated cells (**J**) (n = 8/group). The data are shown as the mean ± SEM. P values were calculated by one-way ANOVA. ^*^p < 0.05, ^**^p < 0.01

To elucidate the role played by GCN5L1 in regulating the CFs phenotype, we modulated GCN5L1 expression by transfecting GCN5L1-interfering (shRNA) and overexpression (LV-GCN5L1) lentiviruses into CFs. Infection of CFs with shRNA-GCN5L1 lentivirus resulted in a 70% reduction in GCN5L1 protein expression. In contrast, CFs infected with LV-GCN5L1 showed a 1.5-fold increase in GCN5L1 expression. We found that GCN5L1 knockdown reversed Ang II-induced fibroblast-to-myofibroblast differentiation, as evidenced by reduced α-SMA, Postn and vimentin expression compared to that of the empty vector controls, and GCN5L1 overexpression facilitated an increase in their expression (Fig. 2A–D). These findings were confirmed by immunofluorescence (Fig. 2E, F). Similar effects were identified with cellular wound healing and transgene migration assays. As shown in Fig. 2G–J, deletion of GCN5L1 inhibited Ang II-induced cell migration, while overexpression of GCN5L1 accelerated cell growth and invasion. These results suggest that GCN5L1 plays an important role in fibroblast-to-myofibroblast differentiation.

### GCN5L1 promotes metabolic shifts during Ang II-induced CFs differentiation

Glucose metabolism alterations have been implicated in fibroblast-to-myofibroblast differentiation [25]. We found that Ang II increased glucose uptake, as evidenced by increases in 2-NBDG fluorescence and Glut1 protein levels (Additional file 1: Fig. S2A–E). To determine the relative contribution of glucose to bioenergetics, we used 2-deoxy-glucose (2-DG) to block the consumption of glucose and identified CF phenotype differences. Inhibition of glucose metabolism by 2-DG reduced Ang II-induced migration and differentiation (Additional file 1: Fig. S2 F, I), without significant effect on the cell viability. Therefore, we suggest that CFs consume glucose as their main energy source and show an increased dependence on glucose during differentiation.

**Fig. 3:**
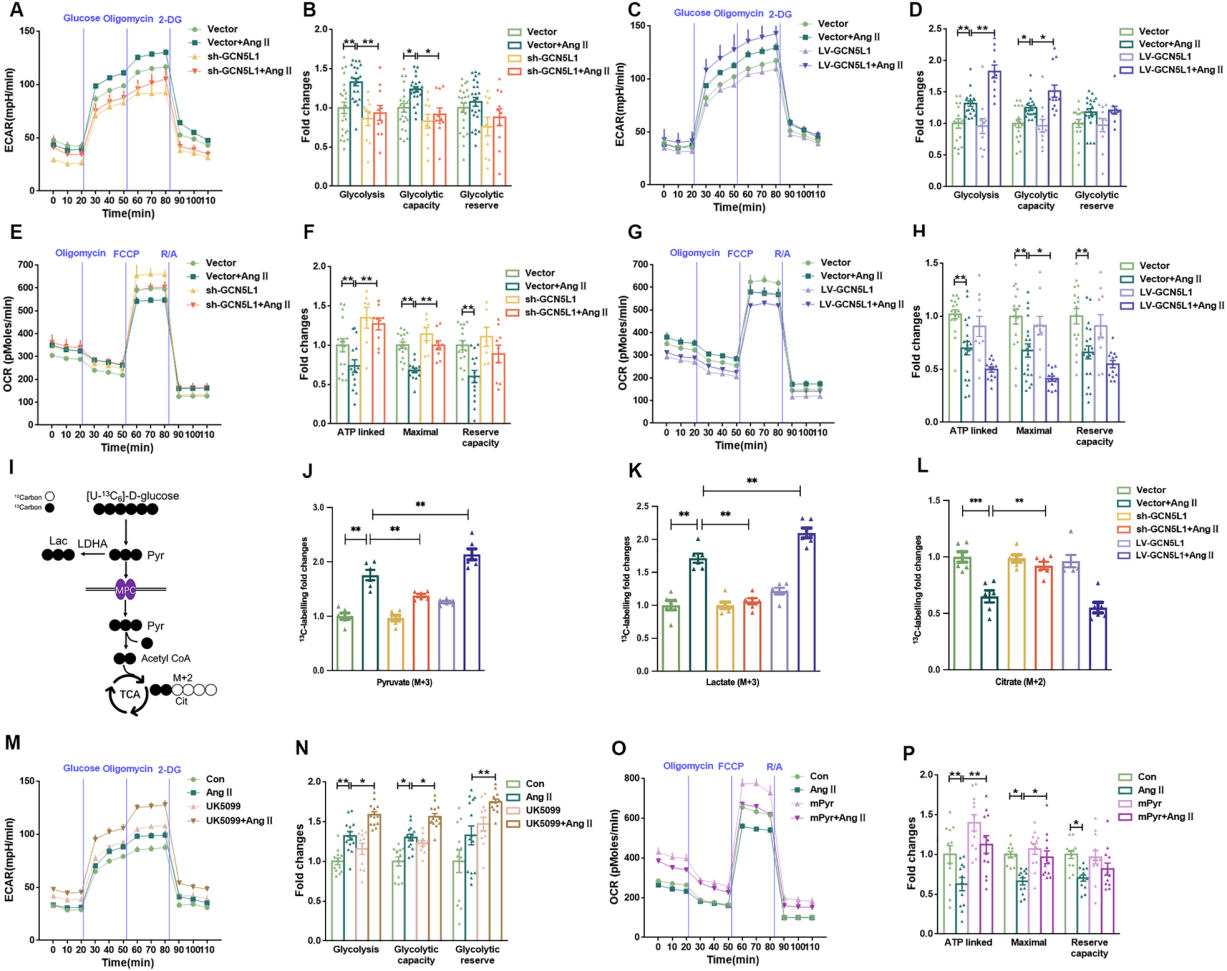
GCN5L1 deficiency restores mitochondrial oxidative phosphorylation. **A **and** B** CFs were treated with or without Ang II after transfection with an empty vector or sh-GCN5L1. The experimental program (**A**) and statistical analysis (**B**) showing the extracellular acidification rate (ECAR) of CFs as measured with a Seahorse XFe96 Extracellular Flux Analyzer (n ≥ 10/group). **C **and** D** CFs were treated with or without Ang II after transfection with an empty vector or LV-GCN5L1. The experimental program (**C**) and statistical analysis (**D**) of the extracellular acidification rate (ECAR) of the CFs as measured with a Seahorse XFe96 Extracellular Flux Analyzer (n ≥ 11/group). **E **and** F** CFs were treated with or without Ang II after transfection with an empty vector or sh-GCN5L1. The experimental program (**E**) and statistical analysis (**F**) of the oxygen consumption rate (OCR) of CFs as measured with a Seahorse XFe96 Extracellular Flux Analyzer (n ≥ 10/group). **G **and** H** CFs were treated with or without Ang II after transfection with an empty vector or LV-GCN5L1. The experimental program (**G**) and statistical analysis (**H**) of the oxygen consumption rate (OCR) of the CFs as measured with a Seahorse XFe96 Extracellular Flux Analyzer (n ≥ 10/group). **I** Schematic diagram showing isotope-tracing experiments. **J**,** K**,** L** CFs were induced differentiation with or without Ang II. [U-^13^C_6_] glucose was added into culture media on 24 h for 30 min. Then, tracing analysis from U-^13^C_6_ glucose was performed. Intracellular abundance of pyruvate (M + 3) (**J**). Intracellular abundance of lactate (M + 3) (**K**). Intracellular abundance of citrate (M + 2) (**L**). Data are the means ± SEM of at least 6 independent experiments. The results are presented as fold increases relative to vector control. ^*^p < 0.05, ^**^p < 0.01. **M** and **N** the experimental program (**M**) and statistical analysis (**N**) of the effect of the extracellular acidification rate (ECAR) on in CFs pre-treated with UK5099 for 1 h then cultured with or without Ang II, which was measured with a Seahorse XFe96 Extracellular Flux Analyzer (n ≥ 11/group). **O **and** P** CFs were pre-treated with methyl pyruvate for 1 h then cultured with or without Ang II. The experimental program (**O**) and statistical analysis (**P**) of the effect of the oxygen consumption rate (OCR) on these cells as measured with a Seahorse XFe96 Extracellular Flux Analyzer (n = 12/group). The data are shown as the mean ± SEM. P values were calculated by one-way ANOVA. ^*^p < 0.05, ^**^p < 0.01

To gain further insights into the impact of GCN5L1 alterations on metabolic changes during CFs differentiation, we performed extracellular acidification rate (ECAR) and oxygen consumption (OCR) analyses. The addition of Ang II increased the glycolytic and glycolytic capacity of CFs (Fig. 3A, B), which was suppressed in GCN5L1-knockdown cells but further elevated in cells with GCN5L1 overexpression (Fig. 3C, D). Moreover, as shown in Fig. 3E, F, Ang II-inhibited ATP-linked and maximal OCR was reversed by GCN5L1 expression knockdown, whereas GCN5L1 overexpression exacerbated this process (Fig. 3G, H). These results suggested that GCN5L1 promoted the Ang II-induced metabolic shift from oxidative phosphorylation to glycolysis.

To examine the metabolic fate of glucose, we performed isotope-tracing experiments with uniformly labelled [U-^13^C_6_]-D-glucose and analysed intracellular metabolites by mass spectrometry. Labelled glucose (M + 6) was expected to produce labelled pyruvate (M + 3) through glycolysis, which would then be converted to lactate (M + 3) in the cytosol or to citrate (M + 2) in mitochondria to fuel tricarboxylic acid (TCA) cycle metabolism (Fig. 3I). After 30 min of incubation with [U-^13^C_6_]-D-glucose, metabolic tracing revealed that the relative abundances of intracellular pyruvate (M + 3) and lactate (M + 3) were significantly increased in the CFs after Ang II stimulation. Whereas, the amount of citrate (M + 2) was decreased. These effects were attenuated by GCN5L1 knockdown, while were further deteriorated by GCN5L1 overexpression (Fig. 3J–L). Neither succinate (M + 2) nor fumarate (M + 2), TCA metabolites downstream of citrate (M + 2), was detected. These results provide direct evidence showing that GCN5L1 exacerbates the impairment to pyruvate metabolism induced by Ang II.

Furthermore, application of UK5099 (an inhibitor of MPC) similarly enhanced both CFs glycolysis and glycolytic capacity (Fig. 3M, N), thereby promoting the fibroblast-to-myofibroblast transition (Additional file 1: Fig. S2J, K). Methyl pyruvate (mPyr), a pyruvate derivative that diffuses freely into mitochondria without relying on carrier-mediated transport, increased the pyruvate content in mitochondria. In contrast, methyl pyruvate-treated fibroblasts showed increased OCR (Fig. 3O, P), which ultimately inhibited the fibroblast-to-myofibroblast differentiation (Additional file 1: Fig. S2L, M).

### GCN5L1 interacts with and acetylates MPC2

**Fig. 4:**
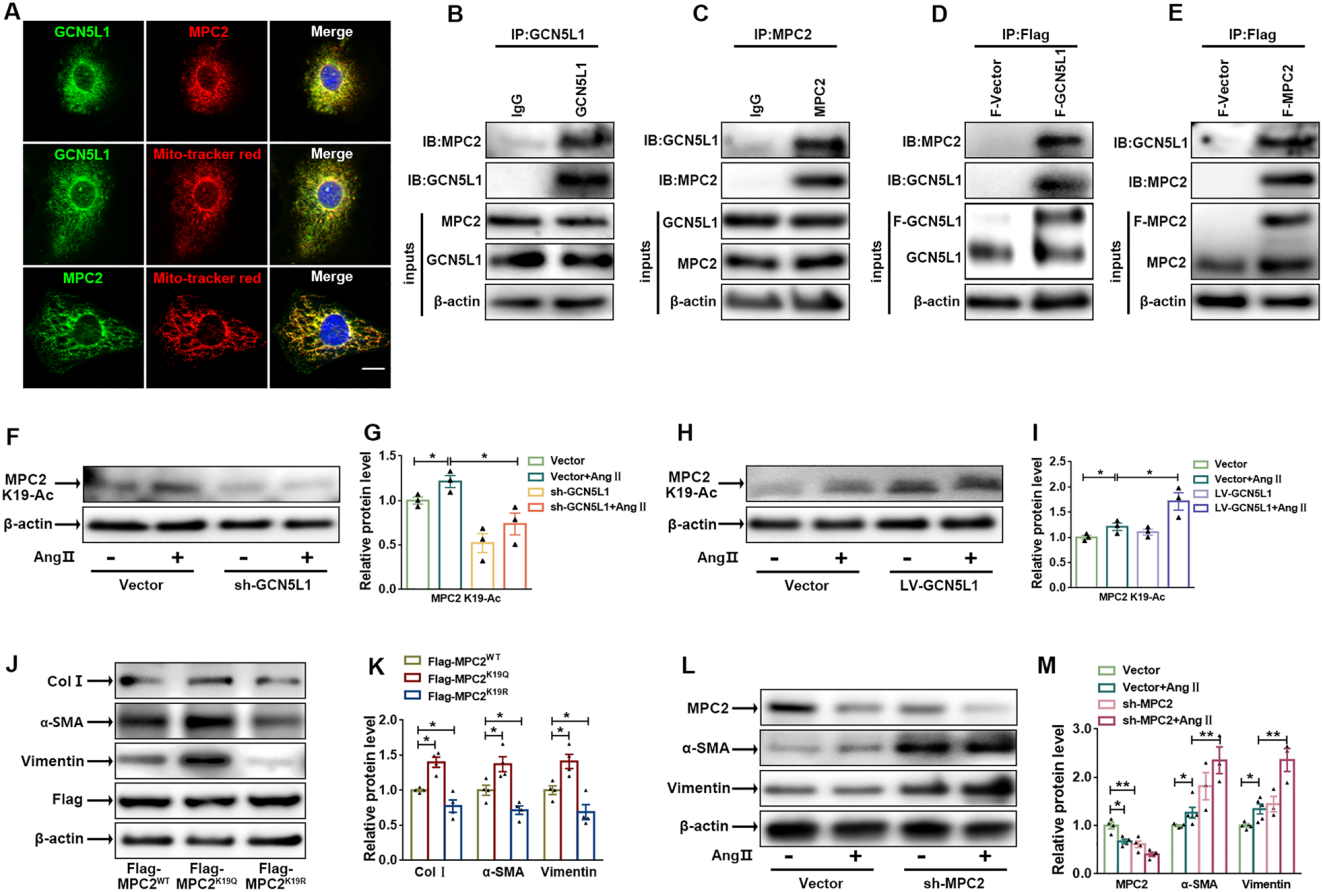
GCN5L1 interacts with and acetylates MPC2. **A** Immunofluorescence assay showing colocalization of GCN5L1 and MPC2 in cellular mitochondria. Scale bar = 50 μm. **B **and** C** Representative western blot images of 293T cell extracts immunoprecipitated with GCN5L1 (**B**) or MPC2 (**C**) and subsequently immunoblotted with anti-MPC2 and anti-GCN5L1 antibodies. **D **and** E** 293T cells transfected with Flag-GCN5L1 (**D**) or Flag-MPC2 (**E**), which was pulled down with anti–Flag beads and subsequently immunoblotted with an anti-GCN5L1 or anti-MPC2 antibody. **F **and** G** immunoblot images showing the Ac-MPC2^K19^ protein expression in CFs treated with Ang II after infection with an empty vector or sh-GCN5L1 (**F**). The quantification of this protein (**G**). n = 3/group. **H **and** I** immunoblot images showing the Ac-MPC2^K19^ protein expression in CFs treated with or without Ang II after infection with an empty vector or LV-GCN5L1 (**H**). The quantification of this protein (**I**). n = 3/group. **J **and** K** Representative immunoblot images showing Col I, a-SMA and vimentin protein expression in CFs transfected with *Flag-MPC2*^*WT*^, *Flag-MPC2*^*K19Q*^ or *Flag-MPC2*^*K19R*^ (**J**) and the quantification of these proteins (**K**) (n = 4/group). **L **and** M** immunoblot images showing MPC2, a-SMA and vimentin protein expression in CFs treated with or without Ang II after transfection with an empty vector or sh-MPC2 (**L**). The quantification of these proteins (**M**) (n ≥ 3/group). The data are shown as the mean ± SEM. P values were calculated by one-way ANOVA. ^*^p < 0.05, ^**^p < 0.01

The transport of pyruvate from the cytoplasm to mitochondria links glycolysis to mitochondrial oxidative phosphorylation (OXPHOS). Pyruvate transport is dependent on the MPC, a heterodimeric complex localized to the inner mitochondrial membrane and comprising two members [26–29]. An increasing number of studies have elucidated the potential role played by MPC2 in cancer cell proliferation and migration [30–33]. PhosphoSitePlus proteomics database (http://www.phosphosite.org) have identified that K19, K26, and K49 as acetylated sites in MPC2 play vital roles in regulating MPC activity. Our mass spectrometry data identified K19 as a potential acetylated site of MPC2 in CFs. To determine the potential molecular mechanisms by which GCN5L1 regulates glucose metabolic reprogramming, we investigated the relationship between GCN5L1 and MPC2. As expected, immunofluorescence showed that GCN5L1 colocalized with MPC2 in cellular mitochondria (Fig. 4A). Consistently, immunoblotting showed that MPC2 and GCN5L1 expression was detected by immunoprecipitation with proteins enriched in targeting GCN5L1 (Fig. 4B) or MPC2 (Fig. 4C), respectively.

In HEK-293T cells transfected with Flag-GCN5L1 (Fig. 4D) or Flag-MPC2 (Fig. 4E) plasmids, the expression of GCN5L1 and MPC2 was detected in proteins pulled down by Flag-beads, confirming that GCN5L1 interacts with both endogenous and exogenous MPC2. To further investigate the site of MPC2 acetylation, we generated an antibody that specifically recognized acetylated MPC2^K19^ by immunizing rabbits with K19-acetylated peptide (AAGARGLRATYHRLLDK(Ac)VEL). Using an acetyl-specific antibody for performing immunofluorescence (Additional file 1: Fig. S3A, B) and western blotting (Fig. 4F–I), we showed that the Ang II-induced increase in Ac-MPC2^K19^ levels was reversed by GCN5L1 knockdown but elevated in GCN5L1-overexpressing cells, indicating that GCN5L1 regulated the acetylation of MPC2^K19^. To determine whether acetylation of MPC2^K19^ mediated MPC activity, 293T cells were transfected with *Flag*-*MPC2*^WT^, *Flag*-*MPC2*^*K19R*^ (the arginine mutant, mimicking lysine deacetylation), and *Flag*-*MPC2*^*K19Q*^ (the glutamine mutant, mimicking lysine 19 acetylation). In the [U-^13^C_3_]-pyruvate sodium tracing assay, pyruvate (M + 3) enters the TCA cycle via MPC, leading to the production of acetyl-CoA (M + 2), which subsequently converts into (M + 2) citrate, α-ketoglutarate (a-KG), succinate, fumarate, and malate (Additional file 1: Fig. S3C). Pyruvate-derived TCA cycle intermediates were more abundant in *MPC2*^*K19R*^ cells compared to *MPC2*^WT^ cells, whereas *MPC2*^*K19Q*^ cells showed the opposite effect (Additional file 1: Fig. S3D), further confirming that K19 acetylation reduces MPC activity. CFs transfected with the *MPC2*^*K19Q*^ plasmid showed an increase in the myofibroblast parameters collagen I, vimentin and α-SMA abundance, and the opposite results were observed for CFs transfected with *MPC2*^*K19R*^ (Fig. 4J, K). MPC2 knockdown promoted the expression of myofibroblast markers, such as α-SMA and vimentin, which were higher than those in the Ang II-treatment group (Fig. 4L, M). Taken together, our data show that hyperacetylation of *MPC2*^*k19*^ leads to MPC inactivation and promotes glycolysis, which ultimately leads to CFs differentiation.

### Increased lactate flux capacity during CFs differentiation

**Fig. 5:**
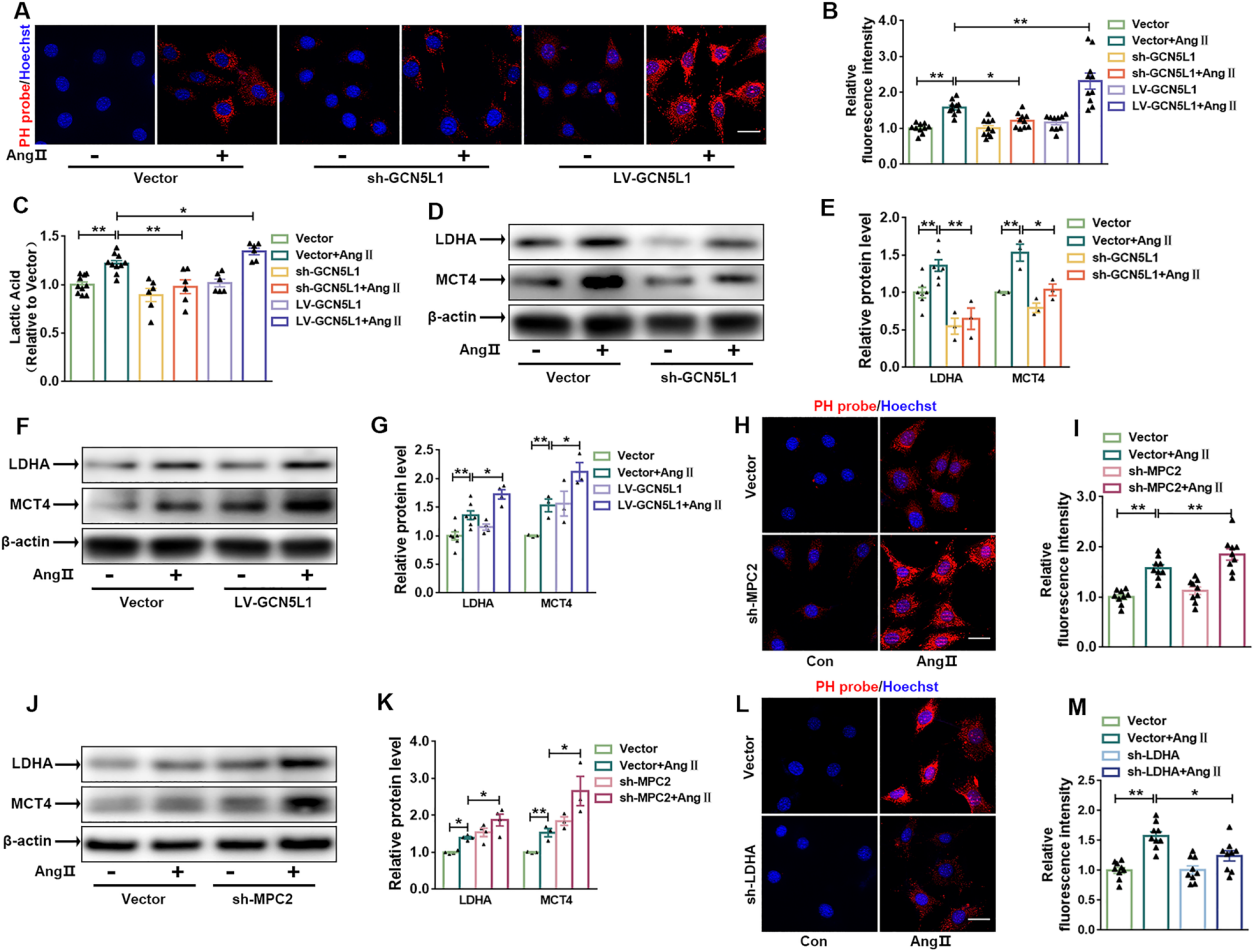
Increased lactate flux capacity during CFs differentiation. **A **and** B**, Images of CFs stained with immunofluorescent pH (potential of hydrogen) probe (red) and Hoechst (blue). Scale bar = 100 μm (**A**). The relative immunofluorescence intensity (**B**) (n = 10/group). **C** Relative quantification of the lactic acid concentration in the cell medium (n ≥ 6/group). **D **and** E** CFs were treated with or without Ang II after transfection with an empty vector or sh-GCN5L1. Representative immunoblot images showing LDHA and MCT4 protein expression (**D**). The quantification of these proteins (**E**) (n ≥ 3/group). **F **and** G** CFs were treated with Ang II after transfection with an empty vector or LV-GCN5L1. Representative immunoblot images showing LDHA and MCT4 protein expression (**F**) (n ≥ 3/group). The quantification of these proteins (**G**). **H **and** I** Representative images of CFs treated with or without Ang II after transfection with an empty vector or sh-MPC2 stained with an immunofluorescent pH probe (red) and Hoechst (blue). Scale bar = 100 μm (**H**). Quantification of the relative fluorescence intensity (**I**) (n ≥ 9/group). **J **and** K** Representative immunoblot images showing LDHA and MCT4 protein expression in CFs treated with or without Ang II after transfection with an empty vector or sh-MPC2 (**J**). The quantification of these proteins (**K**) (n ≥ 3/group). **L **and** M** Representative images of CFs treated with or without Ang II after transfection with an empty vector or sh-LDHA stained with immunofluorescent a pH probe (red) and Hoechst (blue). Scale bar = 100 μm (**L**). Quantification of the relative fluorescence intensity (**M**) (n = 9/group). The data are shown as the mean ± SEM. P values were calculated by one-way ANOVA. *p < 0.05, **p < 0.01

Lactate dehydrogenase A (LDHA) exhibits a high affinity for pyruvate, thereby preferentially converting pyruvate to lactate. As shown in Fig. 5A–C, Ang II increased not only intracellular acidity but also lactate content in the cell medium. These effects paralleled the increase in LDHA and monocarboxylate transporter 4 (MCT4) levels. We suggest that MPC inactivation leads to the accumulation of intracellular lactate, resulting in a decrease in intracellular pH. The high levels of lactate produced were subsequently transported out of the cytoplasm primarily via MCT4, which is the isoform predominant in glycolytic cell realising lactate transport. Furthermore, lack of GCN5L1 in CFs reversed Ang II-induced upregulation of intracellular lactate content, which was accompanied by a decrease in LDHA and MCT4 levels. In contrast, GCN5L1 overexpression further promoted lactate production, leading to increased intracellular acidity (Fig. 5D–G). In cells transfected with MPC2 shRNA, not only increased intracellular acidity (Fig. 5H, I), but also increased the expression of MCT4 compared to that in control cells (Fig. 5J, K). Similarly, we inhibited LDHA levels in CFs using LDHA shRNA and found that LDHA knockdown alleviated the intracellular acidic environment caused by Ang II (Fig. 5L, M).

### Lactate uptake by cardiomyocytes via MCT1

**Fig. 6:**
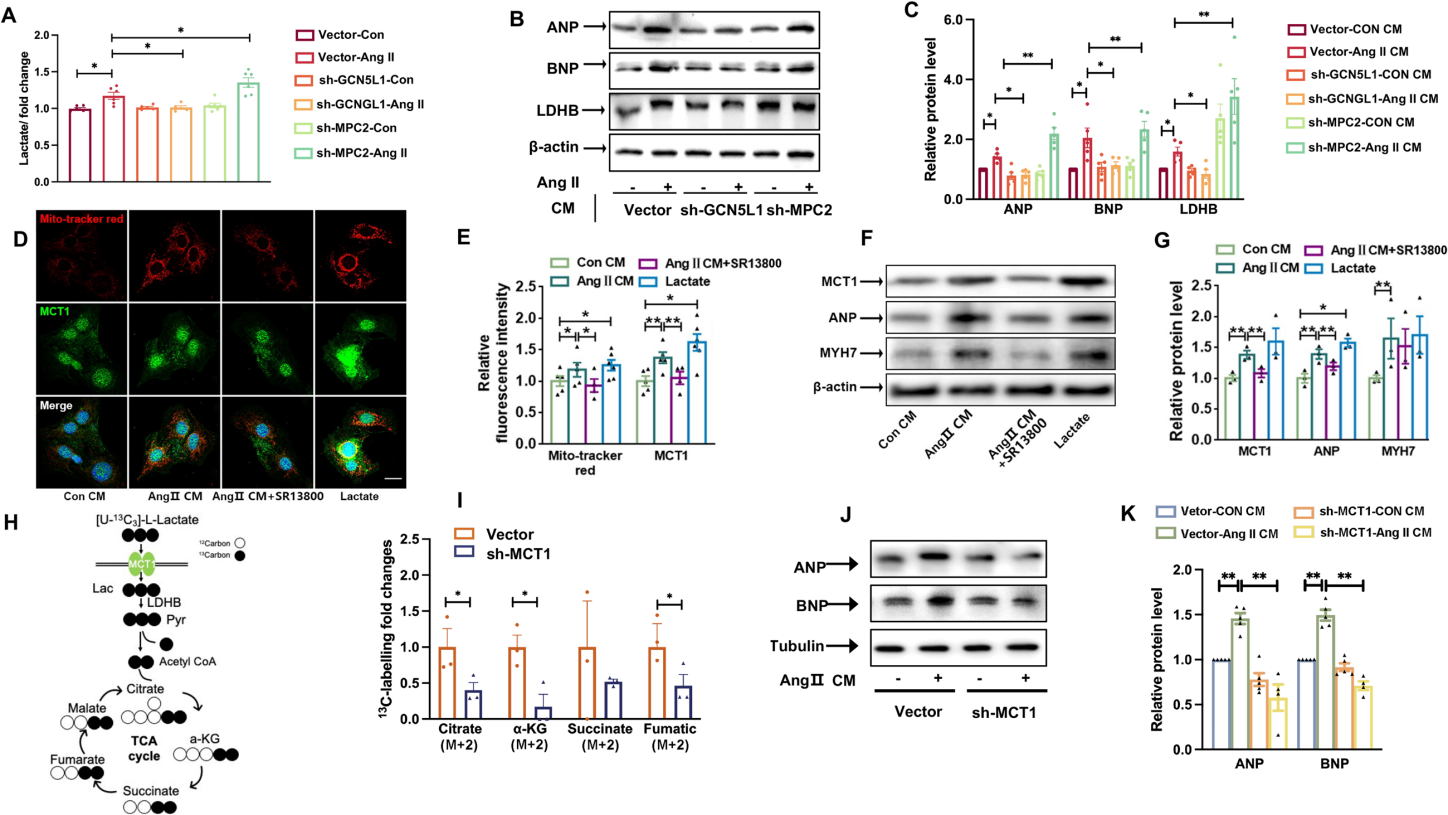
Cardiomyocytes uptake lactate via MCT1. **A** CFs were treated with or without Ang II after transfected with empty vector, sh-GCN5L1 and sh-MPC2 respectively. Relative quantification of the lactic acid concentration in the cell medium was detected n ≥ 4/group. **B **, ** C** Cardiomyocytes were treated with different conditioned medium of CFs cultures. Representative immunoblot images showing LDHB, ANP and BNP protein expression (**B**). The quantification of these proteins (**C**) n = 5/group. ** D**, ** E** Representative images of cardiomyocytes treated with conditioned medium of CFs or lactate (4 × 10^− 3^ mol/L) stained with immunofluorescent Mito Tracker Red (red), MCT1 (green) and DAPI (blue). Scale bar = 50 μm (**D**). Quantification of the relative fluorescence intensity (**E**) (n ≥ 4/group). The data are shown as the mean ± SEM. P values were calculated by one-way ANOVA. ^*^p < 0.05, ^**^p < 0.01. **F **, ** G** Cardiomyocytes were treated with conditioned medium of CFs or lactate. Representative immunoblot images showing MCT1, ANP and MYH7 protein expression (**F**). The quantification of these proteins (**G**) (n = 3/group). **H **, ** I** Schematic diagram showing isotope-tracing experiments (**H**). Cardiomyocytes were infected with empty vector or sh-MCT1. [U-^13^C_3_]-l-lactate was added into culture media for 30 min. Then, tracing analysis from [U-^13^C_3_]-l-lactate was performed. Intracellular abundance of citrate (M + 2), a-KG (M + 2), succinate (M + 2), fumarate (M + 2), Malate (M + 2) was calculated (**I**).** J**, ** K** Cardiomyocytes were infected with empty vector or sh-MCT1, then stimulated with CM of CFs with Ang II treatment for 24 h. Representative immunoblot images showing ANP and BNP protein expression (**J**). The quantification of these proteins (**K**) (n ≥ 4/group). The data are shown as the mean ± SEM. P values were calculated by one-way ANOVA. ^*^p < 0.05,^**^, *p* < 0.01

CFs differentiation is accompanied by enhanced glycolytic flux and the release of more lactate, and we assessed the mechanism by which the amount of lactate in the cardiac microenvironment promotes the hypertrophic phenotype of neighbouring cardiomyocytes. During CFs differentiation into myofibroblasts, Ang II stimulated the release of lactate into the cell supernatant, which we then used to stimulate the cells. Lactate content was further enhanced by MPC2 knockdown, conversely, lactate content was decreased by GCN5L1 knockdown (Fig. 6A). In addition, cardiomyocytes expressed higher ANP, BNP and LDHB after culturing with CMs derived from Ang II-treated CFs compared to controls. This effect was aggravated by incubation with the CM from CFs with MPC2 knockdown, whereas this effect was attenuated by incubation with CM from CFs with GCN5L1 knockdown (Fig. 6B, C).

MCT1 is the predominant isoform in cardiac muscle, which mediates lactate uptake for oxidative metabolism. As shown in Fig. 6D–E, cardiomyocytes were exposed to the CM of CFs treated with or without Ang II, and SR13800 was added to block lactate-induced phenotypic alteration of cardiomyocytes by inhibiting MCT1. Compared with the control group, Ang II CM treatment promoted oxidative stress and MCT1 expression in cardiomyocytes, exhibiting an effect similar to that lactate, and it was reversed by SR13800 administration. Immunoblotting confirmed that increased lactate uptake promotes the expression of MCT1, ANP and MYH7 in cardiomyocytes (Fig. 6F, G), suggesting that excessive lactate secreted by fibroblasts promotes cardiomyocyte hypertrophy. ^13^C_3_-labelled lactate metabolic flux analyses reveal that knockdown MCT1 in cardiomyocytes (Additional file 1: Fig. S4A, B) inhibited lactate-derived TCA cycle intermediates (Fig. 6H, I). While blocking MCT1 expression attenuated the expression of hypertrophy-related genes ANP and BNP exposed to the Ang II CM (Fig. 6J, K).

### Fibroblast-derived lactate controls cardiomyocyte hypertrophy

**Fig. 7:**
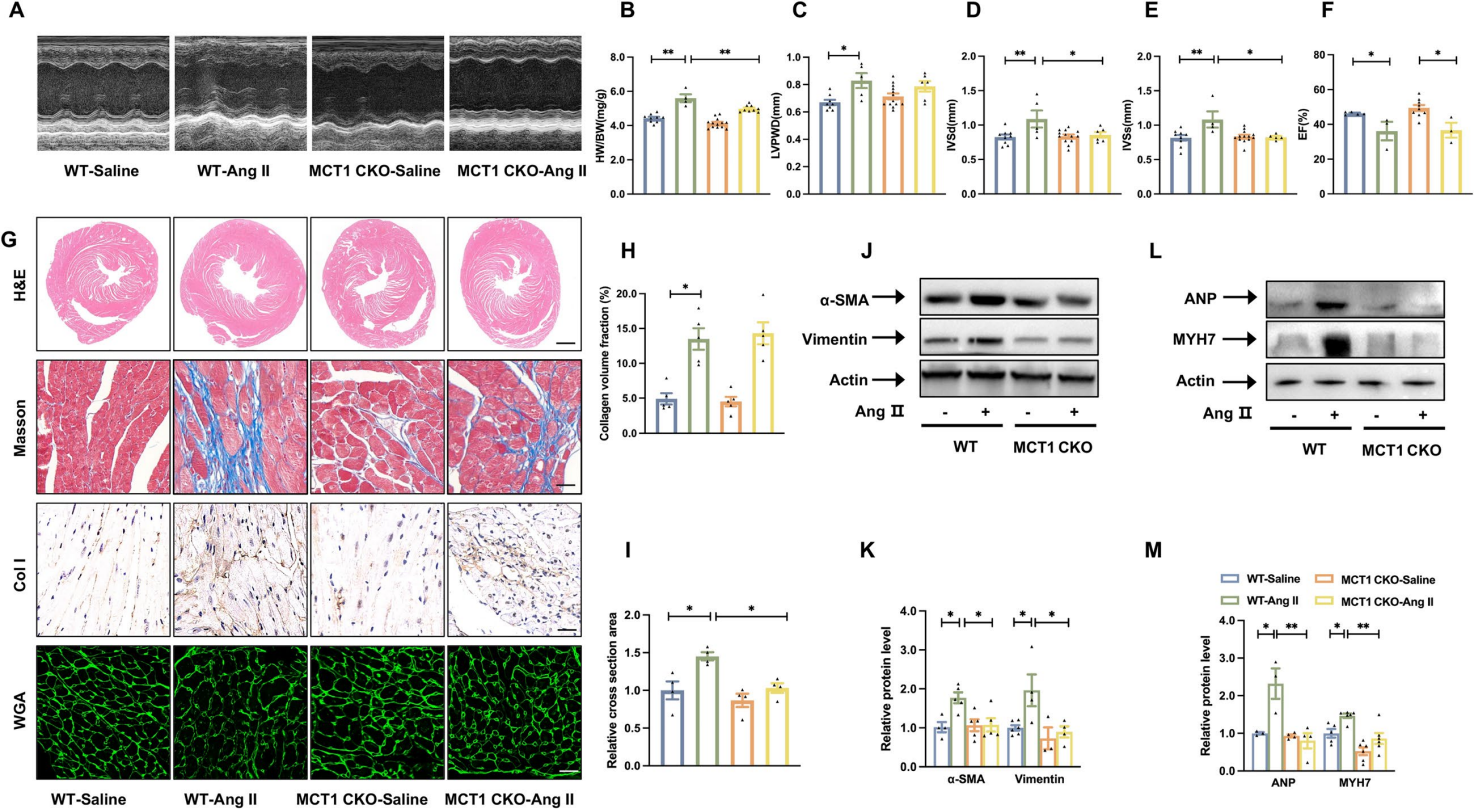
Fibroblast lactate controls cardiomyocyte hypertrophy. **A**, Echocardiographic analysis of WT and MCT1 CKO mice 4 weeks after saline or Ang II infusion. **B** Heart weight/body weight (mg/g) ratio of four groups of mice n ≥ 4/group. **C** through **F** Quantification of echocardiography parameters: LVPWD (**C**), IVSs (**D**) and IVSd (**E**), LVEF (**F**). **G** Representative images of haematoxylin and eosin (H&E) staining, Masson’s staining, immunohistological images of Col I-positive cells in hearts and images after WGA staining. **H** Quantification of collagen volume fraction (n = 5/group). **I** Quantification of the relative cross-sectional area (n = 4/group). **J**, **K**, Representative immunoblot images showing a-SMA and vimentin protein expression in hearts (**J**). The quantification of these proteins (**K**) (n ≥ 3/group). **L**, **M**, Representative immunoblot images showing ANP and MYH7 protein expression in hearts (**L**). The quantification of these proteins (**M**) (n ≥ 3/group). The data are shown as the mean ± SEM. P values were calculated by one-way ANOVA. ^*^p < 0.05, ^**^p < 0.01

Lactate generated in fibroblasts can be transported into cardiomyocytes for utilization by MCT1 [34]. To investigate the effect of lactate shuttling between fibroblasts and cardiomyocytes on cardiac remodelling in vivo, we generated cardiomyocyte-specific MCT1-knockout mice (MCT1 CKO) to establish a hypertension model. The deletion of MCT1 in the mouse cardiomyocytes was confirmed by western blot analysis (Additional file 1: Fig. S4C, D). WT and MCT1 CKO mice were subjected to Ang II treatment for 28 days, although blood pressure was similarly increased in WT and MCT1 CKO mice during Ang II infusion (data not shown). A serial echocardiographic analysis revealed that Ang II increased LVPWD, IVSd and IVSs in WT mice; however, these parameters were significantly attenuated in the MCT1 CKO mice (Fig. 7A, C, D, E). A morphological analysis revealed that, compared with WT mice, MCT1 CKO mice exhibited mitigated cardiac hypertrophy induced by Ang II. Ang II infusion caused an increase in the heart-to-body weight ratio and cardiomyocyte size in the WT mice, which was prevented in the MCT1 CKO mice (Fig. 7B). Despite significant changes in cardiac size, MCT1 deletion had no any effect on LVEF (Fig. 7F). Furthermore, cardiomyocytes-specific MCT1 deletion did not alert Ang II-induced Col I deposition (Fig. 7G, H), it ameliorated cardiomyocyte hypertrophy (Fig. 7G, I). We found that MCT1 knockout reduced the a-SMA and vimentin protein levels 4 weeks after Ang II infusion (Fig. 7J, K), and the levels of the ANP and MYH7 protein were restored (Fig. 7L, M). Therefore, these findings suggest that blocking MCT1 interrupts the fibroblast-to-cardiomyocyte lactate shuttle and may attenuate cardiac remodelling in hypertension. Taken together these results reveal that MCT1 inhibition can reverse and attenuate cellular hypertrophy in cardiomyocytes and normalize mitochondrial oxidative metabolism.

## Discussion

Although cardiac fibroblasts show activity synchronized with that of cardiomyocytes, little is known about the metabolic coupling between cardiac fibroblasts and cardiomyocytes. In the present study, we demonstrated that the intercellular lactate shuttle plays a key role in the regulation of cardiac remodelling during hypertensive stress. The key findings are (i) Ang II elevated lactate production during CFs differentiation into myofibroblasts; (ii) GCN5L1-mediated MPC2^K19^ acetylation reduced MPC activity; (iii) acetylated MPC2^K19^ in CFs promoted glycolysis; and (iv) blocking MCT1 activity disrupted the lactate shuttle, attenuating cardiomyocyte hypertrophy. These results suggest that GCN5L1-mediated MPC2^K19^ is involved in the differentiation of CFs into myofibroblasts, while cardiomyocytes promote hypertrophy through MCT1 uptake of the metabolite lactate.

Metabolic reprogramming has been shown to be critical for the differentiation of CFs into myofibroblasts [35]. However, the molecular basis of this metabolic shift remained unclear. GCN5L1 is a component of the mitochondrial acetyltransferase machinery and plays a key role in regulating mitochondrial protein acetylation and energy metabolism [36]. Consistent with several reports, GCN5L1 levels are increased in cells under metabolic stress, such as during hyperglycaemia or hyperlipidaemia [37, 38]. We found that Ang II increased GCN5L1 levels during the conversion process. Gain- and loss-of-function experiments showed that GCN5L1 exerted a negative effect on the differentiation of CFs. Postn has been shown to be the marker with the highest correlation to the activated CF phenotype [39], and modulation of the activated CF population seems to be a promising approach to prevent adverse cardiac remodelling in response to Ang II. In the present study, we generated GCN5L1^fl/fl^:Postn-Cre mice and confirmed that Ang II-induced collagen and ECM deposition was alleviated in GCN5L1CKO mice. In conjunctions the findings of Lv et al. [40], who reported that loss of GCN5L1 suppressed high-fat diet (HFD)-induced renal lipotoxicity. We concluded that GCN5L1 is negatively correlated with myocardial remodelling in hypertension.

In glucose metabolism, a majority of studies indicated that mitochondrial acetylation levels modulation of the metabolic pathway comports with the finding of increased glycolysis. GCN5L1 promotes PDHA1 acetylation and inhibits pyruvate dehydrogenase (PDH) activity. Therefore, cardiomyocyte-specific deletion of GCN5L1 mice have enhanced cardiac pyruvate oxidation capacity under high fat diet conditions [20]. In contrast, GCN5L1 knockdown cells exhibited higher ECAR rates, suggesting increased glycolysis under aerobic conditions [17, 41]. Therefore, it can be assumed that the effects of GCN5L1-mediated lysine acetylation on glucose oxidase may not be similar in different tissues. In the current study, we found that Ang II-mediated CF differentiation was marked with a glycolytic metabolic signature. Exogenous GCN5L1 increased the Ang II-induced glycolytic capacity and accelerated the accumulation of glycolytic intermediate metabolites. However, loss of GCN5L1 attenuated Ang II-induced glycolytic flux. Moreover, the glycolysis inhibitor 2-DG reduced glycolytic flux and attenuated Ang II-induced transformation of the cellular phenotype. Therefore, we suggest that GCN5L1 may be an important regulator of glycolytic pathways during CFs differentiation.

MPC is a key metabolic branch point that links glycolysis to mitochondrial oxidation [42]. Although Ang II decreased MPC activity, the MPC1 and MPC2 protein levels were unchanged. A previous study has reported that both MPC1 and MPC2 contain acetylated lysine residues and that the acetylation of lysine residues 45 and 46 in MPC1 or lysine 19 and 26 in MPC2 was associated with MPC activity [11, 43]. Our experiment showed that mitochondrial GCN5L1 is the upstream acetylase of MPC2^K19^. Hyperacetylation of MPC2^K19^ not only decreased MPC activity but also increased lactate production and exacerbated Ang II-induced CFs differentiation. These effects were consistent with the suppressive effect of MPC2 shRNA or UK5099 on MPC2 activity. We also showed that this low MPC activity was reversed by increasing the concentration of mPyr, which induces oxidative phosphorylation and inhibits CFs differentiation. We conclude that GCN5L1-mediated acetylation of MPC2^K19^ determined MPC activity.

LDHA is critical for the conversion of pyruvate to lactate, the final step of glycolysis [44]. Due to MPC inactivity, pyruvate can be reduced to lactate in the cytosol by LDHA instead of being transported into the mitochondria. We demonstrate that upregulation of LDHA activity led to increased intracellular acidification of CFs, which is synchronized with transport out of the cytoplasm via MCT4. MCTs are tissue-specific membrane proteins that act as carriers of lactate, pyruvate and ketone bodies with a high degree of substrate specificity. As described, MCT1 has a high lactate affinity and predominates in oxidative cells with high mitochondrial content [45]. In contrast, MCT4 has a lower lactate affinity and tends to accumulate and release lactate from glycolytic cells with less mitochondrial density. MCT1 and MCT4 facilitate the uptake and extraction of lactate, respectively. We found that lactate derived from CFs during differenation to myofibrolasts is released via MCT4, whereas the uptake of exogenous lactate by cardiomyocytes via MCT1, recapitulating the cell‒cell lactate shuttle.

Both neurons and cardiomyocytes have been shown to consume lactate preferentially, even in the presence of glucose, which makes these cells highly sensitive to hypoxia [46]. Interestingly, in the failing human heart, lactate is an important respiratory substrate, and lactate uptake through MCT1 is chronically increased, apparently compensating for decreased fatty acid consumption in mitochondrial energy production. Our experimental results showed that the titres of MCT1 involved in the uptake of lactate and its oxidation to pyruvate were increased in the heart, reflecting lactate consumption by cardiomyocytes. In vitro experiments confirmed that MCT1 knockdown blocked lactate-induced phenotypic alterations of cardiomyocytes by inhibiting MCT1 activity. In addition, cardiac-specific MCT1-knockout mice exhibited reduced ANP and MYH7 levels but partial rescue of Ang II-induced left ventricular hypertrophy. Similar effects have been identified in two other contexts: increased uptake of circulating lactate by cardiomyocytes during exercise or after ischaemic injury [47], inducing proliferation of adult cardiomyocytes in both cases [48]. These findings indicate that the heart depends on exogenous lactate as fuel when cardiac function is elevated.

## Conclusions

CFs contribute to myocardial homeostasis by synthesizing and maintaining the ECM network critical for structural and functional integrity. Ang II not only facilitated CFs proliferation, migratory capacity and collagen synthesis but also increased GCN5L1 expression and promoted hyperacetylation of MPC2^K19^ during CFs differentiation into myofibroblasts. Acetylation modification of MPC2 disrupted mitochondrial pyruvate uptake and accelerated glycolysis, providing the glycolytic metabolite lactate in the microenvironment of the heart. Furthermore, lactate induced phenotypic alteration of cardiomyocytes. Our findings suggest that GCN5L1-MPC2 signalling alters metabolic patterns during Ang II-induced CF differentiation and that blocking MCT1 disrupts the fibroblast-to-cardiomyocyte lactate shuttle and may attenuate cardiac remodelling in hypertension. The tight coupling of the lactate shuttle to metabolism supports the therapeutic potential of CF-cardiomyocyte interactions.

### Research design and methods

The detailed information on the sources of materials used in the experiment in the Major Resources Table (Additional file 2).

#### Animal experiment

GCN5L1^fl/fl^ mice were purchased from Shanghai Model Organisms (Shanghai, China). Periostin-CreERT2 (Postn-Cre) mice were provided by Prof. Bin Zhou (Shanghai Institute of Biochemistry and Cell Biology, Chinese Academy of Sciences). Monocarboxylate transporter 1^fl/fl^ (MCT1^fl/fl^) mice and Myh6-Cre^+/+^ mice were purchased from Cyagen Biosciences Inc. (Suzhou, China). GCN5L1^fl/fl^ mice were bred with Postn-Cre mice to generate Postn-Cre/GCN5L1^fl/fl^ mice (GCN5L1 CKO) and Postn-Cre/GCN5L1^+/+^ mice (WT). MCT1^fl/fl^ mice were bred with Myh6-Cre mice to generate MCT1^fl/fl^/Myh6-Cre mice (MCT1 CKO). Littermates MCT1^fl/fl^ (WT) were used as controls. The animals were housed with a constant temperature (22 °C ± 2 °C) and a 12-h light/dark cycle and allowed free access to food and water.

Eight-week-old male mice were infused with saline or Ang II (1000 ng/kg per minute) for 28 days using a minipump (Alzet, Cupertino, CA) to induce cardiac hypertrophy and fibrosis. GCN5L1 CKO and WT littermates were injected intraperitoneally with tamoxifen (40 mg/kg) for 5 consecutive days starting 1 day after pump implantation. Heart tissues were obtained and stored at − 80 °C or fixed in 4% paraformaldehyde until analysis. All experiments involving the animals were conducted in accordance with the Guide for the Care and Use of Laboratory Animals published by the US National Institutes of Health (NIH Publication, 8th edition, 2011).

### Echocardiography

A high-frequency ultrasound device (Vevo 770, VisualSonics, Canada) was used for ultrasound imaging. Mice were anaesthetized with 3% isoflurane in an induction chamber and maintained with continuous inhalation of a mixture of isoflurane (1.5%) and oxygen. The following parameters were measured in the parasternal short-axis view by 2D M-mode imaging: left ventricular end-diastolic volume (LVEDV), LV end-systolic volume (LVESV), LV posterior wall at end-diastole (LVPWD), LV anterior wall thickness at end-diastole (LVAWD), interventricular septum thickness during diastole (IVSs) and systole (IVSd). Cardiac function was estimated by LV ejection fraction (LVEF), which was derived by the formula EF= (LVEDV-LVESV)/LVEDV × 100%.

### Histological analysis

Heart tissues isolated from euthanized mice were infiltrated with 4% paraformaldehyde, embedded in paraffin and incised into 5-µm-thick sections. The sections were dewaxed with xylene, hydrated with alcohol, and stained with haematoxylin and eosin (H&E) and Masson’s trichrome. For immunohistochemistry (IHC) staining, after antigen retrieval via sodium citrate and blocking with 5% bovine serum albumin (BSA), slides were incubated with collagen I (1:100) overnight at 4 °C, followed by incubation with HRP-conjugated secondary antibody for 60 min. A DAB Substrate kit was used for visualization, and nuclei were stained with haematoxylin. For immunofluorescence (IF) staining, slides were incubated with wheat germ agglutinin (WGA, 1:200) at 4 °C overnight in the dark, and nuclei were stained with DAPI (1:5000). Images were captured with a Zeiss AxioVert A1 microscope (Carl Zeiss). The collagen volume fraction and cross-sectional area were qualified using Image-Pro software.

### Culture of murine cardiac fibroblasts (CFs) and cardiomyocytes

Primary CFs and cardiomyocytes were obtained from C57BL/6J neonatal mice as described previously [49]. Briefly, hearts in 1–3-day-old mice were excised aseptically and only the ventricles were retained. The hearts were maintained in cold Hanks’ balanced salt solution (HBSS) and cut into pieces, subjected to a series of agitation and digestion cycles in HBSS containing collagenase type 2 at 37 °C for 30–35 min. Then, tissues were washed with DMEM and filtered through a 100-µm-cell strainer to remove undigested tissues and to obtain a single-cell suspension. The resulting mixture was centrifuged at 100×*g* for 10 min, and the collected cells were resuspended in Dulbecco’s modified Eagle’s medium (DMEM) with 10% foetal bovine serum (FBS) and 1% penicillin/streptomycin. The single cells obtained were plated onto a 10-cm dish and subjected to differential velocity adherence for 70 min at 37 °C. Cells that adhered were fibroblasts, and nonadherent cells were cardiomyocytes, and thus, CFs were isolated [50]. The CFs were treated with or without 1 × 10^− 6^ mol/L Ang II for 24 h. Conditioned medium (CM) from the CFs culture was harvested for lactate determination or cardiomyocyte stimulation.

### Cell culture and transfection

The cells obtained were cultured in DMEM with 10% FBS and 1% penicillin/streptomycin in an incubator with 5% CO_2_ and 37 °C, and cells in passages 2 or 3 passages were used for experiments. CFs were transfected with lentivirus carrying GCN5L1 short hairpin RNA (shRNA), a GCN5L1-coding sequence (LV-GCN5L1), MPC2 shRNA, LDHA shRNA or an empty vector at a multiplicity of infection of 20. The effect of lentiviral transfection was confirmed by immunoblotting. After infection, CFs were stimulated with or without 1 × 10^− 6^ mol/L Ang II for 24 h, and the CM was harvested for lactic acid determination or cardiomyocyte stimulation. UK5099 (5 × 10^− 5^ mol/L), methyl pyruvate (mPyr, 2 × 10^− 2^ mol/L) or 2-DG (1 × 10^− 2^ mol/L) was added to serum-free medium 1 h before Ang II treatment to identify certain effects. Cardiomyocytes were infected with lentivirus carrying MCT1 short hairpin RNA (shRNA) or an empty vector at a multiplicity of infection of 50. After infection, cardiomyocytes were stimulated with CFs-derived CM for 24 h. HEK-293T cells were maintained in DMEM supplemented with 10% FBS and seeded in 6- or 24-well plates before transfection. Plasmids carrying *Flag-GCN5L1*, *Flag-MPC2*^*WT*^, *Flag-MPC2*^*K19Q*^, and *Flag-MPC2*^*K19R*^ sequences were used to transfect 293T cells with Lipofectamine™ 3000 according to the manufacturer’s instructions.

### Cell wound healing assay

A total of 1 × 10^5^ cells per well were seeded into 6-well plates and cultured in complete medium. When the cells grew to 75% density, the cell monolayer was scratched with a sterile pipette tip in a straight line. After washing three times with PBS to remove cell debris, the cells were treated with or without Ang II and incubated for 24 h. Wound healing in vitro was photographed with a Zeiss AxioVert A1 microscope, and thus, the rate of closure was determined using the following formula: (the wound width at 0 h – the wound width at 24 h)/the wound width at 0 h × 100%.

### Transwell migration and invasion experiments

After starvation of cells for 24 h, 1 × 10^5^ cells in 100 µL serum-free medium per well were seeded into the upper chamber in a Transwell chamber (8-µm pores; Millipore), and 600 µL of complete medium was added to the lower chamber at the same time. After 24 h of incubation, the unmigrated cells on the filter in the upper chamber were removed with a cotton swab, and the cells on the membrane in the lower chamber were fixed with 4% paraformaldehyde for 10 min and stained with 0.1% crystal violet solution for 30 min. The polycarbonate membrane on the Transwell chamber was washed several times with 1×PBS and air-dried. The migrated cells were photographed by inverted fluorescence microscopy.

### Seahorse XF analysis

The extracellular acidification rate (ECAR) and oxygen consumption rate (OCR) of CFs and cardiomyocytes were detected as previously described using a Seahorse XFe96 Analyzer (Seahorse Bioscience, North Billerica, MA, USA) following the manufacturer’s protocol [51]. A Seahorse XF Cell Mito Stress Test Kit and Seahorse XF Glycolysis Stress Test Kit (Agilent Technologies, USA) were employed to measure the OCR and ECAR, respectively. A total of 1 × 10^4^ cells transfected with certain lentiviruses were inoculated in Seahorse 96-well plates with the indicated treatment 1 h before the analysis. The initial medium was replaced with XF assay medium containing 2 × 10^− 3^ mol/L L-glutamine for the ECAR analysis or 2.5 × 10^− 2^ mol/L glucose, 1 × 10^− 3^ mol/L sodium pyruvate, and 2 × 10^− 3^ mol/L L-glutamine for the OCR analysis. For the ECAR assay, nonglycolytic acidification was detected first, and 1 × 10^− 2^ mol/L glucose, 1 × 10^− 6^ mol/L oligomycin, and 5 × 10^− 2^ mol/L 2-deoxy-glucose (2-DG) were injected in sequence to determine the glycolysis, glycolytic and glycolytic reserve capacities. Cell respiration was quantified as the OCR at baseline and following treatment with 1 × 10^− 6^ mol/L oligomycin, 2 × 10^− 6^ mol/L FCCP and 5 × 10^− 7^ mol/L antimycin A/rotenone to calculate ATP-linked OCR, maximal OCR and reserve OC capacity.

### Lactate production assay

The lactate concentration of the culture medium collected from CFs was determined using a lactic acid detection kit (Jiancheng, Nanjing) in accordance with the manufacturer’s instructions. This assay is based on an enzymatic reaction that converts lactate to pyruvate and hydrogen peroxide. The characteristic absorption peak at 530 nm was assessed, and the sample L-lactate concentrations were determined based on the OD values of the samples and standards.

### Immunofluorescence analysis of cells

After Ang II administration, cells were incubated with DMEM containing MitoTracker Red (1:800) or a pH probe (1:500) for 30 min at 37 °C. After washing with 1×PBS, the cells were fixed in acetone and methanol (1:1) at − 20 °C followed by permeabilization with 0.01% Triton X-100. Nonspecific binding sites were blocked with 5% BSA in PBS for 30 min. Cells were incubated with primary antibodies against α-SMA (1:400), GCN5L1 (1:100), MPC2 (1:200) and Ac-MPC2^K19^ (1:100) in 5% BSA at 4 °C overnight. The slides were incubated with the corresponding secondary fluorescently labelled antibodies for 1 h. Nuclei were counterstained with DAPI (1:5000). Samples were examined with a Zeiss AxioVert A1 microscope, and images were analysed with ImageJ software.

#### Immunoprecipitation and coimmunoprecipitation

For immunoprecipitation, 293T cells transfected with *Flag-GCN5L1* or *Flag-MPC2* plasmids and lysed with IP lysis buffer and incubated at 4 °C overnight with anti-Flag M2 agarose. After washing thoroughly in PBS, the beads were eluted with 1× sample buffer and subjected to immunoblot analysis. For coimmunoprecipitation, 293T cells were harvested with IP lysis buffer and allowed to react with immobilized antibodies (GCN5L1, MPC2 and IgG) with gentle mixing at 4 °C overnight using a Pierce Co-Immunoprecipitation (Co-IP) Kit, according to the manufacturer’s instructions. Bound proteins were eluted with reducing SDS lysis buffer, and immunoblotting was performed as described below.

### Western blot analysis

Total proteins were extracted from tissues or cells, and the concentrations were determined with a BCA protein assay kit. Equal amounts of protein were separated on 4–12% SDS–PAGE gels and subsequently transferred to polyvinylidene difluoride (PVDF) membranes. The membranes were blocked with 5% non-fat milk in Tris-buffered saline with 0.1% Tween-20 (TBST) for 1 h at room temperature and incubated with primary antibodies against GCN5L1 (1:500), collagen I (1:1000), α-SMA (1:5000), Postn (1:1000), vimentin (1:1000), ANP (1:500), BNP (1:500), MYH7 (1:500), LDHA (1:1000), MCT1 (1:1000), MCT4 (1:1000), MPC2 (1:1000), Ac-MPC2^K19^ (1:1000), and β-actin (1:8000) at 4 °C overnight. Then, the blots were incubated with corresponding secondary antibodies conjugated with horseradish peroxidase-conjugated (HRP) for 1 h at room temperature. The intensity of the bands was visualized using an enhanced chemiluminescence detection kit and quantified by Quantity One software.

#### Isotope labelling and GC–MS for a metabolic flux analysis

CFs were grown in 10-cm dishes with regular medium until they reached 80% confluence. The cells were then starved in glucose-free medium supplemented with 2.5 × 10^− 2^ mol/L [U-^13^C_6_]-D-glucose (Sigma). 293T cells were transfected with *Flag-MPC2*^*WT*^, *Flag-MPC2*^*K19Q*^, or *Flag-MPC2*^*K19R*^ plasmids. After transfection, cells were cultured in 1% BSA pyruvate-free medium supplemented with 5.5 × 10^− 3^ mol/L glucose, 1 × 10^− 3^ mol/L [U-^13^C_3_]-sodium pyruvate for 30 min. Cardiomyocytes were infected with sh-MCT1 or vector. After infection, cells were cultured in 1% BSA pyruvate-free medium and supplemented with 5.5 × 10^− 3^ mol/L glucose, 4 × 10^− 3^ mol/L [U-^13^C_3_]-sodium lactate for 30 min. Metabolites were assessed in 1 mL of 1:1 water/methanol and subjected to processing via five cycles of 1-min ultrasonication before mixing with ice-cold chloroform. Targeted analysis of citrate levels and labelling was performed by ion-pairing, reverse-phase, ultraperformance liquid chromatography–tandem mass spectrometry on an Agilent 7890 A gas chromatography system coupled to an Agilent 5975 C inert MSD system (Agilent Technologies Inc., CA, USA) [52]. Compounds were identified using an in-house library, with isotopologues corrected on the basis of naturally occurring stable carbons.

### Statistical analysis

Statistical analyses were performed using GraphPad Prism 6.0 (GraphPad Software, Inc., San Diego, CA). All data are presented as the mean ± SEM. The normal distribution of each group was determined by Shapiro–Wilk test, and homogeneity of variance was assessed by Levene’s or Brown-Forsythe tests. Statistical significance between two groups was analysed by unpaired two-tailed Student’s t test. One-way ANOVA followed by Dunnett’s or Tukey’s post hoc tests were used for the comparison of multiple groups. Dunnett’s T3 test was performed for a post hoc analysis of groups with unequal variances. P < 0.05 was considered to indicate statistically significant.

### Supplementary Information

**Additional file 1: Fig. S1.** **A** and **B** WT and GCN5L1CKO mice were subjected to saline or Ang II for 4 weeks. Representative immunoblot images showing GCN5L1 and Postn protein expression in cardiac myofibroblasts isolated from WT or GCN5L1CKO mice (**A**). The systolic blood pressure of WT and GCN5L1CKO mice with saline or Ang II infusion (**B**). The data are shown as the mean ± SEM (n=8/group). P values were calculated by one-way ANOVA.**p <0.01 vs. WT-saline. **Fig. S2.** **A** Immunofluorescence images of CFs stained with 2-NBDG (green) and Hoechst (blue). Scale bar=100 μm. **B** and **C** Representative immunoblot images showing Glut1 protein expression in CFs treated with or without Ang II after infection with an empty vector or sh-GCN5L1 (**B**). The quantification of these proteins (**C**). n≥2/group. **D** and **E** Representative immunoblot images showing Glut1 protein levels in CFs treated with or without Ang II after infection with an empty vector or LV-GCN5L1 (**D**). The quantification of these proteins (**E**). n≥2/group. **F** and **G**, Representative immunoblot images showing α-SMA and vimentin protein expression in CFs pre-treatment with 2-DG then cultured with or without Ang II (**F**). The quantification of these proteins (**G**). n=5/group. **H** and **I** Representative images of the CF wound assay. Scale bar=200 μm (**H**). The percentage of the wound closed (**I**). n=13/group. **J** and **K** Representative immunoblot images showing α-SMA and vimentin protein expression in CFs pre-treated with UK5099 for 1h then cultured with or without Ang II (**J**). The quantification of these proteins (K). n≥3/group. **L** and **M** Representative immunoblot images showing α-SMA and vimentin protein expression in CFs were pre-treated with methyl pyruvate for 1h then cultured with or without Ang II. (**L**). The quantification of these proteins (**M**). n≥3/group. The data are shown as the mean ± SEM. P values were calculated by one-way ANOVA. *p<0.05,**p<0.01. **Fig. S3.** **A** and **B** Immunofluorescence of Ac-MPC2K19 (red) and DAPI (blue) in Ang II treated CFs after transfection with an empty vector, sh-GCN5L1 or LV-GCN5L1. Scale bar = 100 μm (**A**). Quantification of the relative fluorescence intensity (**B**). n = 10/group. **C, **Schematic diagram showing isotope-tracing experiments. **D, **CFs were transfected with *Flag-MPC2WT*, *Flag-MPC2K19Q*or *Flag-MPC2K19R*. [U-13C3]-pyruvate was added into culture media for 30 min. Then, tracing analysis from [U-13C3]-pyruvate was performed. Intracellular abundance of citrate (M+2), α-KG (M+2), succinate (M+2), fumarate (M+2), Malate (M+2) was calculated. n=3/group. The data are shown as the mean ± SEM. P values were calculated by one-way ANOVA. *p <0.05, **p <0.01. **Fig. S4.** **A** Representative immunoblot images showing MCT1 protein expression in cardiomyocytes after infection with an empty vector or sh-MCT1. **B** Quantification of the MCT1 level. n=6/group. The data are shown as the mean ± SEM. P values were calculated by t-test. **p <0.01 vs. vector. **C** Representative immunoblot images showing MCT1 protein expression in the hearts of WT and MCT1CKO mice. **D** Quantification of the MCT1 level. n = 4/group. P values were calculated by t-test. **p <0.01 vs. WT mice.

**Additional file 2:** Major Resources Table

## Data Availability

The authors declare that all supporting data are available within the article and in the supplementary data.
