# Supplementary material for: Fibroblast-to-cardiomyocyte lactate shuttle modulates hypertensive cardiac remodelling
Source: Cell Biosci. 2023 Aug 15;13:151. doi: 10.1186/s13578-023-01098-0 (PMC10426103; doi:10.1186/s13578-023-01098-0)
Supplement: Supplementary file 1 — Additional file 1: Fig. S1. A and B WT and GCN5L1CKO mice were subjected to saline or Ang II for 4 weeks. Representative immunoblot images showing GCN5L1 and Postn protein expression in cardiac myofibroblasts isolated from WT or GCN5L1CKO mice (A). The systolic blood pressure of WT and GCN5L1CKO mice with saline or Ang II infusion (B). The data are shown as the mean ± SEM (n=8/group). P values were calculated by one-way ANOVA.**p <0.01 vs. WT-saline. Fig. S2. A Immunofluorescence images of CFs stained with 2-NBDG (green) and Hoechst (blue). Scale bar=100 μm. B and C Representative immunoblot images showing Glut1 protein expression in CFs treated with or without Ang II after infection with an empty vector or sh-GCN5L1 (B). The quantification of these proteins (C). n≥2/group. D and E Representative immunoblot images showing Glut1 protein levels in CFs treated with or without Ang II after infection with an empty vector or LV-GCN5L1 (D). The quantification of these proteins (E). n≥2/group. F and G, Representative immunoblot images showing α-SMA and vimentin protein expression in CFs pre-treatment with 2-DG then cultured with or without Ang II (F). The quantification of these proteins (G). n=5/group. H and I Representative images of the CF wound assay. Scale bar=200 μm (H). The percentage of the wound closed (I). n=13/group. J and K Representative immunoblot images showing α-SMA and vimentin protein expression in CFs pre-treated with UK5099 for 1h then cultured with or without Ang II (J). The quantification of these proteins (K). n≥3/group. L and M Representative immunoblot images showing α-SMA and vimentin protein expression in CFs were pre-treated with methyl pyruvate for 1h then cultured with or without Ang II. (L). The quantification of these proteins (M). n≥3/group. The data are shown as the mean ± SEM. P values were calculated by one-way ANOVA. *p<0.05,**p<0.01. Fig. S3. A and B Immunofluorescence of Ac-MPC2K19 (red) and DAPI (blue) in Ang II treated CFs after tran [file 13578_2023_1098_MOESM1_ESM.pdf]

# Supplementary Figures

# Fibroblast-to-cardiomyocyte lactate shuttle modulates hypertensive cardiac remodelling

*Tong Wei*[1,2ζ], *Yuetong Guo*[1ζ], *Chenglin Huang*[1], *Mengwei Sun*[3], *Bin Zhou*[4], *Jing Gao*[1], *Weili Shen*[1]

1. Department of Cardiovascular Medicine, State Key Laboratory of Medical Genomics, Shanghai Key Laboratory of Hypertension, Shanghai Institute of Hypertension, Ruijin Hospital, Shanghai Jiao Tong University School of Medicine, Shanghai, 200025, China
2. Department of Cardiology, Shanghai General Hospital, Shanghai Jiao Tong University School of Medicine, Shanghai, 200080, China.
3. Key Laboratory of State General Administration of Sport, Shanghai Research Institute of Sports Science, Shanghai 200030, China
4. New Cornerstone Science Laboratory, State Key Laboratory of Cell Biology, CAS Center for Excellence in Molecular Cell Science, Shanghai Institute of Biochemistry and Cell Biology, Chinese Academy of Sciences, University of Chinese Academy of Sciences, Shanghai, 200031, China

**Running title:** *Cardiac remodelling and lactate shuttle*

[ζ] The authors are contributed equally to this work.

**Correspondence should be addressed to:*

Weili Shen, PhD

Department of Cardiovascular Medicine, State Key Laboratory of Medical Genomics,

Shanghai Key Laboratory of Hypertension, Shanghai Institute of Hypertension, Ruijin

Hospital, Shanghai Jiao Tong University School of Medicine, Shanghai, China

Tel: 0086-21-64314015

Fax: 0086-21-64314015

E-mail：weili_shen@hotmail.com or wlshen@sibs.ac.cn

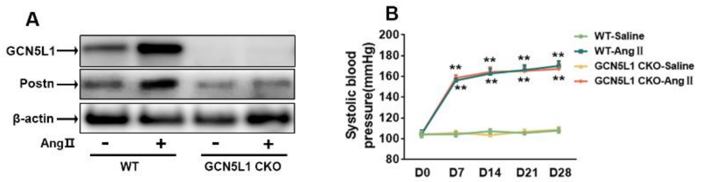


Figure S1

***Figure S1*** **A** and **B,** WT and GCN5L1CKO mice were subjected to saline or Ang II for 4 weeks. Representative immunoblot images showing GCN5L1 and Postn protein expression in cardiac myofibroblasts isolated from WT or GCN5L1CKO mice (**A**). The systolic blood pressure of WT and GCN5L1CKO mice with saline or Ang II infusion (**B**). The data are shown as the mean ± SEM (n=8/group). P values were calculated by one-way ANOVA. $^{**}p<0.01$ vs. WT-saline.

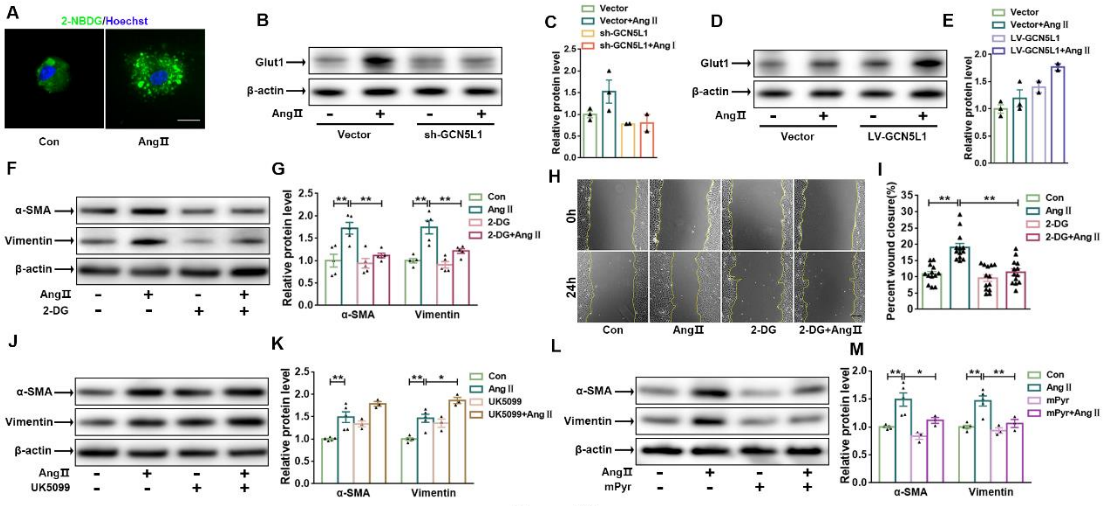


**Figure S2**

***Figure S2*** **A,** Immunofluorescence images of CFs stained with 2-NBDG (green) and Hoechst (blue). Scale bar=100 μm. **B and C,** Representative immunoblot images showing Glut1 protein expression in CFs treated with or without Ang II after infection with an empty vector or sh-GCN5L1 (**B**). The quantification of these proteins (**C**). n⩾2/group. **D and E,** Representative immunoblot images showing Glut1 protein levels in CFs treated with or without Ang II after infection with an empty vector or LV-GCN5L1 (**D**). The quantification of these proteins (**E**). n ⩾2/group. **F and G,** Representative immunoblot images showing α-SMA and vimentin protein expression in CFs pre-treatment with 2-DG then cultured with or without Ang II (**F**). The quantification of these proteins (**G**). n=5/group. **H and I,** Representative images of the CF wound assay. Scale bar=200 μm (**H**). The percentage of the wound closed (**I**). n=13/group. **J and K,** Representative immunoblot images showing α-SMA and vimentin protein expression in CFs pre-treated with UK5099 for 1h then cultured with or without Ang II (**J**). The quantification of these proteins (**K**). n⩾3/group. **L and M,** Representative immunoblot images showing α-SMA and vimentin protein expression in CFs were pre-treated with methyl pyruvate for 1h then cultured with or without Ang II. (**L**). The quantification of these proteins (**M**). n⩾3/group. The data are shown as the mean ± SEM. P values were calculated by one-way ANOVA. $^{*}p<0.05$, $^{**}p<0.01$.

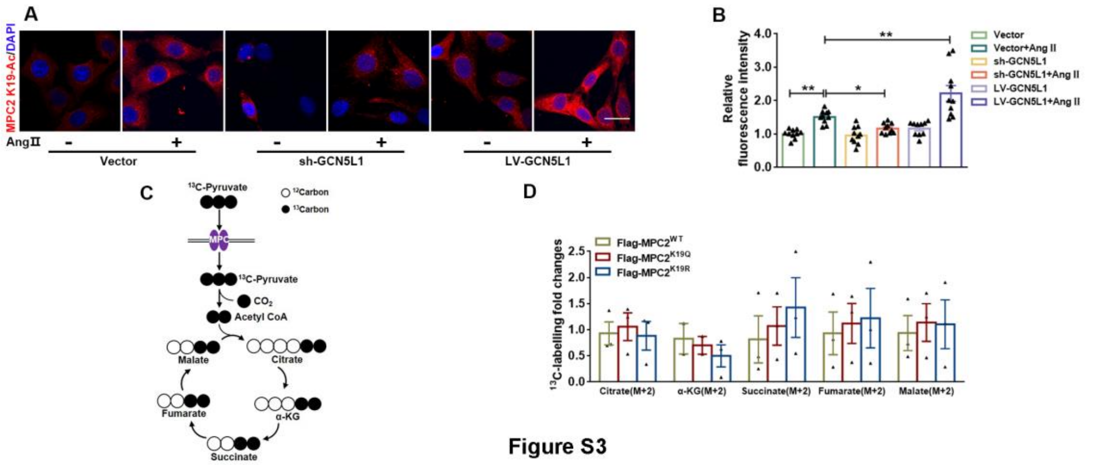


***Figure S3*** **A** and **B**, Immunofluorescence of Ac-MPC2K19 (red) and DAPI (blue) in Ang II treated CFs after transfection with an empty vector, sh-GCN5L1 or LV-GCN5L1. Scale bar=100 μm (A). Quantification of the relative fluorescence intensity (B). n=10/group. **C,** Schematic diagram showing isotope-tracing experiments. **D,** CFs were transfected with *Flag-MPC2$^{WT}$*, *Flag-MPC2$^{K19Q}$* or *Flag-MPC2$^{K19R}$*. [U-$^{13}C_3$]-pyruvate was added into culture media for 30 min. Then, tracing analysis from [U-$^{13}C_3$]-pyruvate was performed. Intracellular abundance of citrate (M+2), α-KG (M+2), succinate (M+2), fumarate (M+2), Malate (M+2) was calculated. n=3/group. The data are shown as the mean ± SEM. P values were calculated by one-way ANOVA. *$p<0.05$, **$p<0.01$.

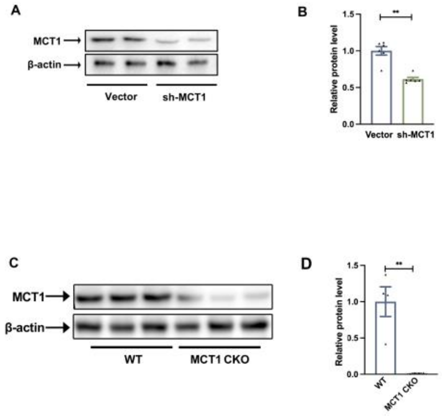


Figure S4

***Figure S4 A,*** Representative immunoblot images showing MCT1 protein expression in cardiomyocytes after infection with an empty vector or sh-MCT1. ***B,*** Quantification of the MCT1 level. n=6/group. The data are shown as the mean ± SEM. P values were calculated by *t-test*. $^{**}p<0.01$ *vs*. vector. **C,** Representative immunoblot images showing MCT1 protein expression in the hearts of WT and MCT1CKO mice. **D**, Quantification of the MCT1 level. n=4/group. P values were calculated by t-test. $**p<0.01$ vs. WT mice.
