# Supplementary material for: Fibroblast-to-cardiomyocyte lactate shuttle modulates hypertensive cardiac remodelling
Source: Cell Biosci. 2023 Aug 15;13:151. doi: 10.1186/s13578-023-01098-0 (PMC10426103; doi:10.1186/s13578-023-01098-0)
Supplement: Supplementary file 2 — Additional file 2: Major Resources Table [file 13578_2023_1098_MOESM2_ESM.pdf]

Major Resources Table
Fibroblast-to-cardiomyocyte lactate shuttle modulates hypertensive cardiac remodelling

Tong Wei[1,2ζ], Yuetong Guo[1ζ], Chenglin Huang[1], Mengwei Sun[3], Bin Zhou[4], Jing Gao[1], Weili Shen[1]

1. Department of Cardiovascular Medicine, State Key Laboratory of Medical Genomics, Shanghai Key Laboratory of Hypertension, Shanghai Institute of Hypertension, Ruijin Hospital, Shanghai Jiao Tong University School of Medicine, Shanghai, 200025, China
2. Department of Cardiology, Shanghai General Hospital, Shanghai Jiao Tong University School of Medicine, Shanghai, 200080, China.
3. Key Laboratory of State General Administration of Sport, Shanghai Research Institute of Sports Science, Shanghai 200030, China
4. New Cornerstone Science Laboratory, State Key Laboratory of Cell Biology, CAS Center for Excellence in Molecular Cell Science, Shanghai Institute of Biochemistry and Cell Biology, Chinese Academy of Sciences, University of Chinese Academy of Sciences, Shanghai, 200031, China

Running title: *Cardiac remodelling and lactate shuttle*

[ζ] The authors are contributed equally to this work.

**Correspondence should be addressed to:*

Weili Shen, PhD

Department of Cardiovascular Medicine, State Key Laboratory of Medical Genomics, Shanghai Key Laboratory of Hypertension, Shanghai Institute of Hypertension, Ruijin Hospital, Shanghai Jiao Tong University School of Medicine, Shanghai, China

Tel: 0086-21-64314015

Fax: 0086-21-64314015

E-mail：weili_shen@hotmail.com or wlshen@sibs.ac.cn

Animals (in vivo studies)

Species	Vendor or Source	Background	Sex
GCN5L1$^{flox/flox}$	Shanghai Model Organisms	C57BL/6J	Male/Female
Periostin-CreERT2	Professor Bin Zhou<br>Shanghai Institute of Biochemistry and Cell Biology	C57BL/6J	Male/Female
MCT1$^{flox/flox}$	Cyagen Biosciences Inc	C57BL/6J	Male/Female
Myh6-Cre	Cyagen Biosciences Inc	C57BL/6J	Male/Female

Reagents

Name	Source	Catalog	Working Concentration
Angiotensin II (Ang II)	Calbiochem	05-23-0101	1000 ng/kg/min (in vivo)<br>1×10^{-6} mol/L (in vitro)
Tamoxifen	Sigma-Aldrich	T5648	100 mg/kg
Coelenterazine	YEASEN	40905ES02	5×10^{-6} mol/L
[U-$^{13}C_6$]-D-glucose	Sigma-Aldrich	310808	2.5×10^{-2} mol/L
Sodium l-lactate-$^{13}C_3$	Santa Cruz	Sc-364293	4×10^{-3} mol/L
Sodium pyruvate-$^{13}C_3$	Sigma-Aldrich	490717	1×10^{-3} mol/L
WGA	Invitrogen	W6748	5 μg/mL
PH-probe	Invitrogen	P35372	5×10^{-6} mol/L
Seahorse XF Glycolysis Stress Test Kit	Agilent Technologies	103020-100	
The Seahorse XF Cell Mito Stress Test Kit	Agilent Technologies	103015-100	
Lactic Acid assay kit	Nanjing Jiancheng	A019-2-1	
Fetal bovine serum (FBS)	Gibco	10099141	
Dulbecco modified Eagle medium (DMEM)	Gibco	11965092	
Mito-tracker red	Invitrogen	M7513	1×10^{-6} mol/L
2-NBDG	Sigma-Aldrich	72987	1×10^{-4} mol/L
UK5099	Sigma-Aldrich	PZ0160	5×10^{-5} mol/L
Methyl pyruvate	Sigma-Aldrich	371173	2×10^{-2} mol/L
SR13800	Calbiochem	509663	1×10^{-7} mol/L

Antibodies

Target antigen	Source	Catalog	Working concentration
GCN5L1	Invitrogen	PA5-41425	1×10^{-6}g/ml
Vimentin	CST	5741S	1×10^{-6}g/ml
Periostin	Invitrogen	PA5-34641	1×10^{-6}g/ml
Collagen I	Sigma-Aldrich	Ab21286	1×10^{-6}g/ml

α-SMA	Sigma-Aldrich	A2547	1×10^{-6}g/ml
ANP	Santa Cruz	Sc-20158	1×10^{-6}g/ml
BNP	Santa Cruz	Sc-18817	1×10^{-6}g/ml
LDHA	CST	3582T	1×10^{-6}g/ml
MCT4	Santa Cruz	Sc-376140	1×10^{-6}g/ml
MPC2	CST	46141S	1×10^{-6}g/ml
Ac-MPC2^{K19}	HuaBio	HAPM0406-AC	1×10^{-6}g/ml
Glut1	Thermo	PA1-46152	1×10^{-6}g/ml
MCT1	Novus	NBP1-59656	1×10^{-6}g/ml
MYH7	Santa Cruz	Sc-53090	1×10^{-6}g/ml
LDHB	Beyotime	AF7365	1×10^{-6}g/ml
